# A Biocompatible Cinchonine‐Based Catalyst for the CO_2_ Valorization into Oxazolidin‐2‐ones Under Ambient Conditions

**DOI:** 10.1002/chem.202500473

**Published:** 2025-03-26

**Authors:** Lucia Invernizzi, Caterina Damiano, Emma Gallo

**Affiliations:** ^1^ Department of Chemistry University of Milan Via C. Golgi 19 Milan 20133 Italy

**Keywords:** atmospheric conditions, aziridines, CO_2_ valorization, organocatalysts, oxazolidinones

## Abstract

A metal‐free, biocompatible catalyst for the cycloaddition of CO_2_ to *N*‐alkyl aziridines was easily obtained by protonating the natural and nontoxic alkaloid (+)‐cinchonine. This bifunctional catalytic system promoted the synthesis of the desired products under very mild experimental conditions (room temperature and atmospheric CO_2_ pressure) and without the aid of any cocatalyst. No specific equipment is required, making the procedure practical for application in any laboratory. The high synthetic value of this methodology can be attributed to the combination of excellent regioselectivity in oxazolidinone synthesis and the remarkable chemical stability of the catalyst, which can be recycled and reused for at least three consecutive cycles without any significant loss of activity.

## Graphical abstract

A simple derivative of the natural alkaloid (+)‐cinchonine promoted the cycloaddition of CO_2_ to aziridines at room temperature and atmospheric CO_2_ pressure without the aid of any cocatalyst. Oxazolidin‐2‐ones were obtained in high yields and regioselectivities employing a highly accessible procedure that does not require any specific equipment.

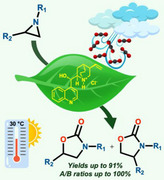

## 1 Introduction

The continuous increase of global temperature is a major concern for today's society. Recently, 2024 has been recorded as the first year with an average temperature overcoming the threshold of 1.5 °C above the preindustrial level.^[^ ^1^ ^]^ In order to achieve the challenging target of net zero emissions of greenhouse gases in 2050, strategies to mitigate climate change are even more urgent.^[^ ^2^ ^]^ In this context, interest toward carbon capture, utilization and storage (CCUS) is continuously growing,^[^ ^3^, ^4^, ^5^ ^]^ as CO_2_ represents an inexpensive and abundant source of carbon atoms and its conversion into valuable compounds is an attractive way to convert a waste into a resource.^[^ ^6^ ^]^

Up to now, numerous strategies employing carbon dioxide as a C1 building block for the synthesis of chemicals, including both reductive^[^ ^7^, ^8^, ^9^ ^]^ and nonreductive^[^ ^10^, ^11^, ^12^ ^]^ transformations, have been reported. Generally, the use of CO_2_ as a starting material is practicable only with the aid of catalysts that allow to overcome the intrinsic stability of this molecule (Δ*H*_f_° = −393.5 kJ mol^−1^)^[^ ^13^ ^]^ by altering its symmetry and linearity.^[^ ^14^, ^15^, ^16^, ^17^ ^]^

**Scheme 1:**
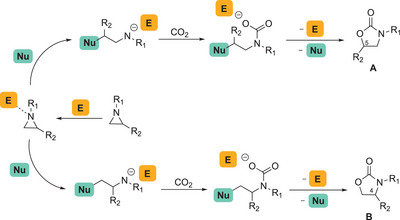
General mechanism of the CO_2_ cycloaddition to aziridines forming oxazolidin‐2‐ones.

Among products achievable through nonreductive conversion of CO_2_, oxazolidin‐2‐ones deserve attention due to their wide applications as chiral auxiliaries,^[^ ^18^, ^19^ ^]^ pharmaceuticals,^[^ ^20^, ^21^ ^]^ and synthetic intermediates.^[^ ^22^ ^]^ The cycloaddition of CO_2_ to aziridines, being a 100% atom‐efficient strategy, represents an attractive method for the eco‐compatible synthesis of these compounds.^[^ ^12^ ^]^ The abovementioned reaction follows the general mechanism reported in Scheme 1, in which an electrophilic (E) and a nucleophilic (Nu) species play a synergic action that is essential for a productive reaction outcome. Indeed, the nucleophilic attack to one of the aziridine carbon atoms, that paves the way for the subsequent reaction with CO_2_, becomes more favorable after the activation of the substrate performed by the electrophile. Depending on the carbon atom attacked by nucleophilic species, two different regioisomers of the final oxazolidinone product (**A** and **B**) can be formed, and the ratio is dependent on steric and electronic characteristics of both the aziridine and the catalyst.

Recently, a great attention has been focused on the development of both homogeneous^[^ ^23^, ^24^ ^]^ and heterogeneous^[^ ^25^, ^26^, ^27^ ^]^ bifunctional catalysts able to promote the process of interest. Considering that both E and Nu species are displayed by the same molecule, the presence of additives or cocatalysts is not required with a consequent enhance of the reaction sustainability. However, the use of a bifunctional catalyst does not necessarily determine an improvement of the reaction sustainability, since the overall synthetic strategy required for producing the catalyst as well as the nature of reaction conditions (CO_2_ pressure, temperature, catalytic loading, eco‐tolerability of the reaction components), must also be taken into a great account.^[^ ^12^ ^]^

Due to the interest toward low‐toxic synthetic procedures, the use of eco‐friendly bifunctional organocatalysts has become largely attractive. The activity of salts obtained from commercially available organic bases (e.g., pyridine and DABCO) was demonstrated.^[^ ^28^, ^29^ ^]^ However, these systems required harsh reaction conditions (temperatures up to 100 °C and CO_2_ pressures up to 6.0 MPa) and their applicability is further limited by the toxicity of the starting materials. In this view, natural α‐amino acids could be an appealing alternative due to their biocompatible nature,^[^ ^30^, ^31^ ^]^ but once again the desired oxazolidin‐2‐ones could be obtained only by employing very high CO_2_ pressures (up to 8.0 MPa) and long reaction times (up to 36 h). Finally, all the above mentioned catalysts have been used in solvent‐free conditions, restricting the reaction scope to liquid substrates and requiring high amounts of aziridines, whose synthesis is time‐consuming.

In this context, our group has recently discovered the catalytic activity of TPPH_4_Cl_2_ (**1**) (TPP = dianion of tetraphenyl porphyrin) and other metal‐free protonated porphyrins,^[^ ^32^ ^]^ easily prepared by treating the commercially available TPPH_2_ with mineral or organic acids. TPPH_4_Cl_2_ promoted the synthesis of *N*‐alkyl oxazolidin‐2‐ones at 100 °C and 1.2 MPa of CO_2_ pressure, leading to satisfactory yields (95%–100%) and **A** regioselectivities (95%–100%), despite the low catalytic loading employed (1.0 mol%). The reaction mechanism, studied via DFT calculations, revealed the synergic activation of both the aziridine and CO_2_ reagents by the catalyst. The bifunctional TPPH_4_Cl_2_ supplied both the active nucleophilic chloride anion, essential for the ring‐opening step, and the tetrapyrrolic porphyrin platform, which plays a crucial role in inducing the observed reaction regioselectivity.

**Figure 1:**
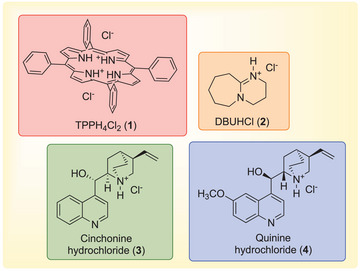
Chemical structures of the tested catalysts.

In order to better evaluate the influence of the electrophilic portion of the catalyst in driving the reaction outcome, we decided to carry on the study of the catalytic activity of other bifunctional organocatalytic systems, bearing the same nucleophilic moiety (Cl^−^) and different electrophilic scaffolds, all endowed with a NH^+^ group. Among the various possibilities, we have selected compounds represented in Figure 1, which can be easily obtained from cheap and commercially available starting materials.

Since, 1,8‐diazabicyclo[5.4.0]undec‐7‐enium chloride (DBUHCl, **2**) was recently found effective in promoting the cycloaddition of CO_2_ to epoxides under mild conditions,^[^ ^33^ ^]^ it seemed interesting to explore its activity also toward aziridines. On the other hand, the use of protonated *Cinchona* alkaloids **3** and **4** appeared attractive in view of both the biocompatible nature of these compounds and the presence of a chiral platform. It is worth noting that the benign and pharmaceutical nature of *Cinchona* alkaloids derivatives^[^ ^34^, ^35^, ^36^ ^]^ not only faces up concerns about acceptable levels of catalyst traces in drug substances but also offers the possibility for creating binary pharmaceuticals. In such cases, the final therapeutic effect could be due to the combination of the two different active substances: the oxazolidinone and the *Cinchona* catalyst.

Even if chiral oxazolidin‐2‐ones have numerous applications,^[^ ^19^, ^37^ ^]^ only limited examples of their synthesis via cycloaddition of CO_2_ to aziridines have been reported to date.^[^ ^38^, ^39^, ^40^, ^41^ ^]^ Furthermore, to the best of our knowledge, the use of chiral catalysts for synthesizing enantiopure oxazolidin‐2‐ones from racemic aziridines has never been described. Therefore, considering the wide use of *Cinchona* alkaloids derivatives in asymmetric catalysis,^[^ ^42^, ^43^, ^44^ ^]^ the choice of catalysts **3** and **4** had also the aim of exploring their capability to perform enantioselective CO_2_ cycloadditions to aziridines.

In this study, we first evaluated the catalytic activity of compounds **2**–**4** in the oxazolidin‐2‐ones synthesis, also comparing their performances with that of the previously studied TPPH_4_Cl_2_ (**1**).^[^ ^32^ ^]^ After selecting the best catalyst, the reaction conditions were optimized, and the reaction scope explored. In addition, preliminary experiments to hypothesize a plausible catalytic mechanism were also performed.

## 2 Results and Discussion

TPPH_4_Cl_2_ (**1**) and DBUHCl (**2**) were synthesized by employing reported procedures.^[^ ^32^, ^45^ ^]^ Conversely, a new synthetic pathway was developed for the synthesis of cinchonine hydrochloride (**3**) and quinine hydrochloride (**4**). These two compounds were obtained by adding a H_2_O/THF solution of HCl (37% in water) to the THF solution of the corresponding alkaloid. The products were easily separated by filtration and obtained in yields higher than 90% without further purifications. The complete characterization of compounds **3** and **4** is reported in the Experimental Section. On the other hand, quinine hydrochloride dihydrate (**5**), tested for evaluating the influence of water on the reaction outcome, was commercially available and used as received. The cycloaddition of 1‐butyl‐2‐phenylaziridine to CO_2_ forming 3‐butyl‐5‐phenyloxazolidin‐2‐one (**6**) was employed as the model reaction and it was performed even in the presence of HCl (37% in water) alone. This test was fundamental to assess the advantages of employing catalysts **1**–**5** rather than using a simple and cost‐effective hydrochloric acid.

According to the experimental conditions optimized in our previous work^[^ ^32^ ^]^ for the TPPH_4_Cl_2_ (**1**)‐catalyzed reactions, tests reported in Table 1 were initially run for 16 h at 100 °C with 1.2 MPa of CO_2_, 1 mol% of catalytic loading and by using *^i^* PrOH as the reaction solvent (Table 1, entries 1a–6a). Under these experimental conditions, catalysts **1**–**5** were all effective in promoting the formation of **6**. Complete conversion of the substrate was achieved only in the presence of TPPH_4_Cl_2_ (**1**), which showed instead the worst selectivity and **A**/**B** ratio (Table 1, entry 2a), probably due to the incomplete solubility of the protonated porphyrin in *^i^* PrOH. Catalysts **2**–**5** gave significantly better results in terms of selectivity (Table 1, entries 3a–6a), and the best outcome was achieved in the reaction promoted by **2** (Table 1, entry 3a), even if 100% of selectivity has never been reached due to the contemporary formation of a mixture of the 1,4‐dibutyl‐2,5‐diphenylpiperazine isomers as the reaction byproducts (see Supporting Information). It is worth noting that, under these experimental conditions, HCl (37% in water) formed the desired compounds with the non‐negligible 71% of aziridine conversion, 82% of selectivity and the **A**/**B** ratio of 90:10. Consequently, the use of more sophisticated catalysts under harsh reaction conditions raises some doubts, highlighting the importance of always comparing the activity of new catalysts with that of simple, standard molecules to justify their practical application.

**Table 1:** Synthesis of 3‐butyl‐5‐phenyloxazolidin‐2‐one (**6**) promoted by catalysts **1**–**5** under different reaction conditions.^[^ ^a^ ^]^

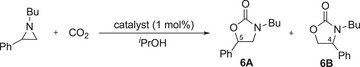					
Entry	Catalyst	Conversion (%^[^ ^b^ ^]^)	Selectivity (%^[^ ^b^ ^]^)	Yield (%^[^ ^b^ ^]^)	6A/6B Ratio^[^ ^b^ ^]^
1a^[^ ^c^ ^]^	HCl (37% aq)	71 (39)^[^ ^f^ ^]^	82 (95)^[^ ^f^ ^]^	58 (37)^[^ ^f^ ^]^	90:10 (90:10)^[^ ^f^ ^]^
1b^[^ ^d^ ^]^		90	87	78	95:5
1c^[^ ^e^ ^]^		0	–	0	–
2a^[^ ^c^ ^]^	TPPH_4_Cl_2_ (**1**)	100 (100)^[^ ^f^ ^]^	73 (100)^[^ ^f^ ^]^	73 (100)^[^ ^f^ ^]^	91:9 (95:5)^[^ ^f^ ^]^
2b^[^ ^d^ ^]^		64	87	57	96:4
2c^[^ ^e^ ^]^		0	–	0	–
3a^[^ ^c^ ^]^	DBUHCl (**2**)	86	95	82	95:5
3b^[^ ^d^ ^]^		31	94	29	97:3
3c^[^ ^e^ ^]^		5	80	4	96:4
4a^[^ ^c^ ^]^	Cinchonine hydrochloride (**3**)	83	93	77	91:9
4b^[^ ^d^ ^]^		57	89	51	97:3
4c^[^ ^e^ ^]^		20	55	11	95:5
5a^[^ ^c^ ^]^	Quinine hydrochloride (**4**)	80	89	71	92:8
5b^[^ ^d^ ^]^		39	92	36	97:3
5c^[^ ^e^ ^]^		17	71	12	95:5
6a^[^ ^c^ ^]^	Quinine hydrochloride dihydrate (**5**)	82	90	74	92:8
6b^[^ ^d^ ^]^		24	88	21	97:3
6c^[^ ^e^ ^]^		5	80	4	100:0

^[a]^ Reactions were stirred for 16 h in 0.5 mL of *^i^* PrOH.

^[b]^ Calculated by ^1^H NMR spectroscopy by using 2,4‐dinitrotoluene as the internal standard.

^[c]^ 100 °C and 1.2 MPa of CO_2_ pressure.

^[d]^ 30 °C and 1.2 MPa of CO_2_ pressure.

^[e]^ 30 °C and 0.1 MPa of CO_2_ pressure (plastic balloon).

^[f]^ 1,2‐Dichloroethane (DCE) as the reaction solvent.

On the other hand, due to the importance of developing energy‐saving processes, the catalytic activity of compounds **1**–**5** were explored under milder experimental conditions, maintaining the CO_2_ pressure at 1.2 MPa and reducing the temperature to 30 °C (Table 1, entries 1b–6b). Apart from HCl (Table 1, entry 1b), all the catalysts yielded lower conversion values, with a less pronounced decrease in case of **3** (Table 1, entry 4b). The best conversion was still achieved with **1** (Table 1, entry 2b), whereas **2** remained the optimal system in terms of reaction selectivity (Table 1, entry 3b). Comparable **A**/**B** ratios were obtained with all the catalysts under these conditions.

In view of the still good results obtained under these conditions in the presence of HCl (37% aq.) and to perform an ideal “green” process, experiments were repeated by working at 30 °C and atmospheric CO_2_ pressure (Table 1, entries 1c–6c). These extremely mild reaction conditions determined a complete loss of effectiveness of HCl and **1** (Table 1, entries 1c and 2c) and a strong reduction of the **2** and **5** catalytic activities (Table 1, entries 3c and 6c). Under these conditions, the presence of water seemed to have a negative influence on the reaction outcome, since **4** showed better performances than **5** (Table 1, entries 5c and 6c). Catalysts **3** and **4** provided encouraging results, the former in terms of conversion (Table 1, entry 4c), the latter in terms of selectivity (Table 1, entry 5c). Therefore, it seemed worth to further explore the activity of cinchonine hydrochloride (**3**) and quinine hydrochloride (**4**) at 30 °C and 0.1 MPa, considering both the biocompatible nature of the two compounds and the attractiveness of the employed experimental conditions. It is worth noting that the importance to optimize the catalytic performances of **3** and **4** is strongly related to the limited number of organocatalysts that can effectively promote cycloaddition of CO_2_ to aziridines under eco‐friendly and ambient conditions.^[^ ^12^ ^]^

**Figure 2:**
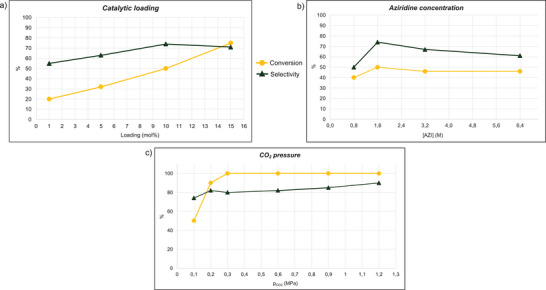
Study of the reaction dependence on (a) catalytic loading, (b) aziridine concentration, and (c) CO_2_ pressure.

Given the cost‐effectiveness and low toxicity of **3**, the reaction productivity should be improved by increasing the catalytic loading, without compromising the sustainability of the process. Therefore, the synthesis of **6** was run at 30 °C and atmospheric CO_2_ pressure in the presence of 5 mol%, 10 mol% and 15 mol% of **3** (Figure 2a and Table S1 in Supporting Information). The highest aziridine conversion (75%) was achieved with a catalytic loading of 15 mol%. However, the best selectivity (74%) and **A**/**B** ratio (99:1) were obtained in the presence of 10 mol% of catalyst **3**, albeit a lower aziridine conversion (50%) was observed. Only a modest reaction efficiency was observed by using 5 mol% of **3**.

Quinine hydrochloride (**4**) was also tested at 10 mol% of catalytic loading, and the synthesis of **6** was obtained with selectivity and **A**/**B** ratio worse than those achieved in the presence of **3** (see Table S1 in Supporting Information). Consequently, the latter compound was selected as the target catalyst to optimize all the other reaction conditions in order to maximize the reaction productivity.

Even though no significant effects on the reaction outcome were observed by testing five different 1‐butyl‐2‐phenyl aziridine concentrations (Figure 2b and Table S2 in Supporting Information), 1.6 M was elected as the best value due to the lowest formation of 1,4‐dibutyl‐2,5‐diphenylpiperazine, which can be formed as side‐products by the **3**‐catalyzed aziridine dimerization (see Supporting Information).

Then, the effect of CO_2_ pressure was explored (Figure 2c and Table S3 in Supporting Information). Although complete aziridine conversion was achieved at CO_2_ pressures above 0.2 MPa, 100% reaction selectivity toward the desired product **6** was never obtained, even at 1.2 MPa CO_2_ pressure. Considering the environmental advantages of conducting reactions under atmospheric CO_2_ pressure, we explored the possibility of improving reaction performance by replacing *^i^* PrOH with alternative solvents and extending the reaction time, rather than using pressurized CO_2_.

**Figure 3:**
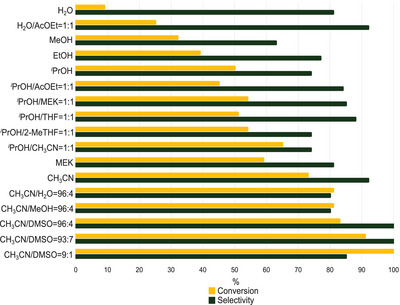
Study of the reaction dependence on the solvent.

Several reaction solvents were then applied (Figure 3 and Table S4 and Figure S4 in Supporting Information). Since **3** is a water‐soluble organocatalyst, the model reaction was first run in H_2_O or in a biphasic H_2_O/AcOEt mixture. Unfortunately, unsatisfactory results were reached, particularly in terms of aziridine conversion. Therefore, other polar protic solvents, in which **3** also showed good solubility, were examined in combination with a cosolvent to guarantee a good CO_2_ solubility^[^ ^46^, ^47^, ^48^ ^]^ in the reaction medium (see Supporting Information). The analysis of several solvent mixtures revealed that a complete aziridine conversion was achieved with CH_3_CN/DMSO = 9:1, whereas 100% selectivity for **6** was reached by employing CH_3_CN/DMSO = 96:4 or 93:7. Considering the importance of avoiding the formation of side products to maximize the reaction sustainability, the latter solvent mixture was finally chosen as the reaction medium also in view of the best compromise between conversions and selectivities.

The collected data highlight a strong impact of the solvent composition, revealing that both CO_2_ and the catalyst need to be well dissolved in the reaction medium in order to achieve good results. Indeed, the best outcomes were obtained by mixing CH_3_CN, in which CO_2_ is high soluble,^[^ ^48^ ^]^ and DMSO, that improves the solubilization of the polar catalyst **3**. The same concept helps to rationalize the observed detrimental effect of water, since this solvent does not ensure a good balance between CO_2_ and catalyst solubility.^[^ ^49^ ^]^ In addition, the strong solvation of the Cl⁻ anion by H₂O hampers its nucleophilic attack on the aziridine substrate,^[^ ^50^ ^]^ with consequent negative results.

Employing CH_3_CN/DMSO = 93:7 as the solvent, the formation of **6** over time was monitored via IR spectroscopy in order to detect the optimal reaction time, and 10 h seemed sufficient to reach the maximum conversion in case of the CO_2_ cycloaddition of 1‐butyl‐2‐phenylaziridine (see Table S5 and Figure S5 in Supporting Information). However, since the model substrate is highly reactive, the substrate scope was performed by running reactions for 16 h, in order to achieve appreciable conversions also for less reactive substrates.

**Scheme 2:**
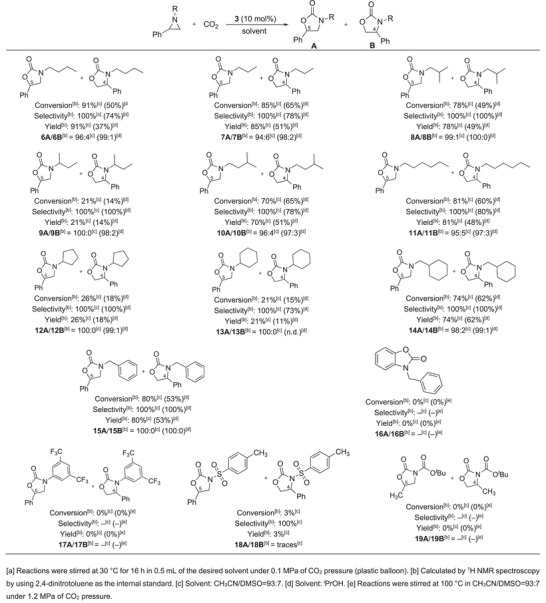
Study of the reaction scope in the presence of cinchonine hydrochloride (**3**).^(a)^

As reported in Scheme 2, catalyst **3** was efficient in promoting the cycloaddition of carbon dioxide to different *N*‐alkyl aziridines under ambient temperature and CO_2_ pressure. The analysis of results revealed that the length of the linear alkyl group linked to the nitrogen atom had no strong influence on the reaction outcome, since comparable conversions and selectivities were observed for the synthesis of oxazolidin‐2‐ones **6A**/**6B** (*^n^* Bu), **7A**/**7B** (*^n^* Pr) and **11A**/**11B** (*n*‐hexyl). Conversely, the reaction productivity decreased in the presence of both branched and cyclic *N*‐substituents, revealing the negative effect of high steric hindrance around the aziridine nitrogen atom on the catalytic performance. Even if compounds **9A**/**9B** (*N*‐*sec*‐butyl), **12A**/**12B** (*N*‐cyclopentyl) and **13A**/**13B** (*N*‐cyclohexyl) were obtained with 100% of selectivity and very good **A**/**B** ratio, aziridine conversions up to 26% were registered. The negative effect of the steric hindrance around *N*‐aziridine atom can be minimized by introducing a ─CH_2_─ spacer between the nitrogen atom and the bulky group, as observed for the synthesis of products **8A**/**8B** and **14A**/**14B**. The introduction of the methylene bridge between the nitrogen atom and the cyclohexyl substituent was responsible for the increase of the reaction yield from 21 to 74% (compare synthesis of **13A** and **14A**).

Then, the effect of the electronic characteristics of the *N*‐substituted group was analyzed. The reaction performed well for *N*‐benzyl derivatives and compound **15A** was obtained in 80% yield as the only regioisomer. On the other hand, no‐conversion was observed when 1‐(3,5‐bis(trifluoromethyl)phenyl)‐2‐phenyl aziridine or 1‐(Boc)‐2‐methylaziridine were reacted with CO_2_ (synthesis of compounds **17** and **19**). The same negative result was achieved when a bicyclic aziridine was employed as the substrate and the formation of compound **16** was not observed.

Considering the generally less pronounced reactivity of *N*‐aryl, *N*‐acyl and bicyclic aziridines with respect to *N*‐alkyl aziridines, the reactions were also performed in harsher conditions (100 °C, 1.2 MPa), but again no traces of desired products were observed.

Finally, only traces of 3‐tosyl‐5‐phenyloxazolidin‐2‐one (**18**) was detected when the corresponding aziridine was employed as the substrate.

To better evaluate the synthetic potential of the reported procedure, it was essential to assess the chemical stability of the organocatalyst **3**. Thus, the synthesis of compound **6** was repeated three consecutive times in the presence of the original amount of the catalyst. The desired product was obtained with an overall yield of 84%, 100% selectivity, and a 95:5 **A**/**B** ratio, demonstrating that the catalyst recycling does not compromise the catalytic activity. Moreover, the chemical stability of **3** under the employed reaction conditions was also proved by treating the catalyst under the optimized experimental condition (CH_3_CN/DMSO = 93:7, 16 h, 30 °C, 0.1 MPa of CO_2_ pressure) in the absence of 1‐butyl‐2‐phenylaziridine substrate. The ^1^H NMR analysis of the reaction mixture did not reveal either traces of (+)‐cinchonine or other decomposition products.

Finally, it is interesting to underline that the pre‐catalyst (+)‐cinchonine can be easily regenerated at the end of a catalytic cycle by a simple procedure. First, the reaction crude was treated with a H_2_O/CH_2_Cl_2_ mixture and then, the addition of a diluted aqueous NaOH solution to the aqueous phase was responsible for the precipitation of (+)‐cinchonine as a white solid, which was recovered by a filtration in 75% yield.

Even if the synthesis of 3‐butyl‐5‐phenyloxazolidin‐2‐one **6A** could, in principle, be enantioselective due to the presence of the chiral catalyst **3**, the ${\left[\alpha \right]}_{D}$ analysis of the isolated product revealed the formation of a racemic mixture. Next, the enantiopure (*S*)‐1‐benzyl‐2‐octylaziridine (**20**) was synthesized (see Supporting Information) in order to evaluate the ability of **3** to promote a stereospecific transformation. Unfortunately, when **20** was reacted with CO_2_ in the presence of **3**, the formation of corresponding oxazolidinone was not observed, even when the CO_2_ pressure was increased to 1.2 MPa.

**Figure 4:**
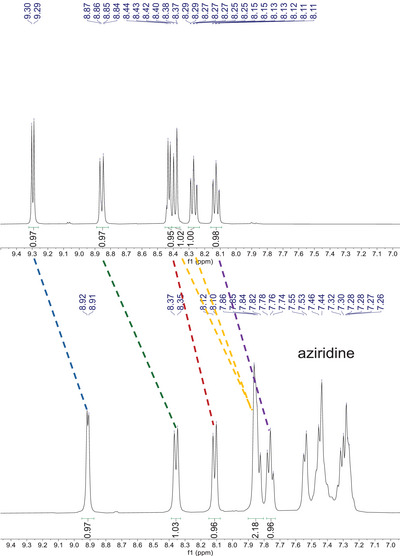
Shift of the aromatic signals of **3** after formation of the adduct with 1‐butyl‐2‐phenylaziridine.

To shed some light on the reaction mechanism, the reaction of catalyst **3** toward either 1‐butyl‐2‐phenyl aziridine or CO_2_ was analyzed by ^1^H NMR spectroscopy. While collected data showed no reaction between **3** and CO_2_, an appreciable shift of the aromatic signals of both the aziridine and the catalyst was observed when the two compounds were mixed in CD_3_OD (Figure 4) suggesting the formation of an adduct between **3** and the aziridine. The formation of such an adduct was also suggested by the increased catalyst solubility, which was always observed upon the addition of the substrate to a suspension of **3** in the reaction medium.

When the CD_3_OD solution of the so‐obtained adduct was saturated with CO_2_ at RT for 16 h, the formation of compound **6** was observed (Figure S6–S8 in Supporting Information). However, since the substrate conversion was not complete, the signals of the adduct between **3** and the aziridine were still detected. A more detailed mechanistic study, including a DFT analysis of the catalytic cycle, is now under investigation to suggest how the CO_2_ activation occurs and a plausible mechanism of the **3**‐catalyzed process.

## 3 Conclusion

A convenient method for the synthesis of *N*‐alkyl oxazolidin‐2‐ones via cycloaddition of CO_2_ to aziridines has been developed. In the presence of 10 mol% of the biocompatible catalyst cinchonine hydrochloride (**3**), the desired products were achieved in satisfactory yields under ambient experimental conditions (30 °C, 0.1 MPa of CO_2_). The metal‐free system **3** was easily obtained by employing a rapid and efficient procedure and, due to its bifunctional nature, it was very efficient in the absence of any additive or cocatalyst. In addition, its high chemical stability allowed performing three subsequent catalytic cycles without significant loss of activity. Good regioselectivities (up to 100%) were observed, revealing that even simple and cheap organocatalysts can efficiently drive the reaction selectivity.

Due to the mild conditions employed and the eco‐compatible nature of the catalyst, the reported strategy aligns with the societal request for sustainable processes *for the conversion of waste into resources* and has the potential for a future scale‐up and practical applications. It is worth noting that the reported procedure can be carried out in any laboratory, as it does not require specialized equipment (e.g., autoclaves), making the valorization of CO_2_ into useful chemicals highly accessible.

For a further increase of sustainability, the use of an appropriate solid support to support cinchonine hydrochloride (**3**) is currently under investigation to perform the reaction under heterogeneous conditions, favoring the recovery and reuse of the catalyst.

## 4 Experimental Section

### 4.1 General Methods

Unless otherwise specified, solvents were commercial grade and used as received. TPPH_4_Cl_2_ (**1**) was synthesized according to the procedure reported in our previous work^[^ ^32^ ^]^ and aziridines were synthesized by following reported strategies.^[^ ^51^, ^52^, ^53^, ^54^ ^]^ Synthesis and characterization of the previously unreported (*S*)‐1‐benzyl‐2‐octylaziridine (**20**) are described in the Supporting Information. All the other starting materials were commercial products and used as received.

NMR spectra (Figures S9–S38 in Supporting Information) were recorded at room temperature either on a Bruker Avance 300‐DRX, operating at 300 MHz for ^1^H and at 75 MHz for ^13^C, or on a Bruker Avance 400‐DRX spectrometers, operating at 400 MHz for ^1^H and at 101 MHz for ^13^C. Chemical shifts (ppm) are reported relative to TMS. ^1^H NMR signals of the compounds described in the following were attributed by 2D NMR techniques. Assignments of the resonances in ^13^C NMR were made by using the APT pulse sequence, HSQC and HMBC techniques. The following abbreviations have been used for NMR signals: s = singlet, d = doublet, t = triplet, m = multiplet, dd = doublet of doublets, pt = pseudo triplet, and bs = broad singlet. Infrared spectra were recorded on a Varian Scimitar FTS 1000 spectrophotometer. UV–vis spectra were recorded on an Agilent 8453E instrument. Elemental analyses and mass spectra were recorded in the analytical laboratories of Milan University. A Jasco P‐1030 polarimeter was employed for [α]_D_ measurements, which were conducted at 589 nm using a 1 mL cell with a length of 1 dm.

### 4.2 Synthesis of Cinchonine Hydrochloride (**3**)

A 37% aqueous solution of HCl (0.092 mL, 1.1×10^−3^ mol) was diluted with THF (1.0 mL) and added dropwise under stirring to a (+)‐cinchonine (0.25 g, 8.5×10^−4^ mol) THF (85 mL) solution. A white solid precipitated and after concentrating the solvent to half of the starting volume, the solid was collected in a filter, washed three times with Et_2_O and dried under vacuum at 50 °C for 6 h (92% yield).

${\left[\alpha \right]}_{D}^{22}$ = +173.5° (3.0×10^−3^ M, MeOH). ^1^H NMR (400 MHz, CD_3_OD): δ 9.27 (d, *J* = 5.6 Hz, 1H, H_2’_), 8.81 (d, *J* = 8.5 Hz, 1H, H_8’_), 8.40 (d, *J* = 5.5 Hz, 1H, H_3’_), 8.36 (d, *J* = 8.5 Hz, 1H, H_5’_), 8.26 (pt, *J* = 7.6 Hz, 1H, H_6’_), 8.12 (pt, *J* = 7.6 Hz, 1H, H_7’_), 6.60 (d, *J* = 15.2 Hz, 1H, H_9_), 6.20–6.11 (m, 1H, H_10_), 5.31 (pt, *J* = 10.0 Hz, 2H, H_11_), 4.30–4.24 (m, 1H, H_6A_), 3.79 (pt, *J* = 9.0 Hz, 1H, H_8_), 3.66–3.56 (m, 2H, H_2_), 3.42–3.35 (m, 1H, H_6B_), 2.81 (q, *J* = 8.5 Hz, 1H, H_3_), 2.53 (pt, *J* = 11.0 Hz, 1H, H_7A_), 2.07–1.99 (m, 2H, H_5_), 1.93–1.85 (m, 1H, H_4_), 1.33–1.29 ppm (m, 1H, H_7B_). ^13^C NMR (101 MHz, CD_3_OD): δ 158.9 (C_10’_), 144.4 (C_2’_), 137.9 (C_4’_), 136.6 (C_10_), 134.8 (C_6’_), 130.7 (C_7’_), 125.8 (C_9’_), 124.7 (C_8’_), 121.3 (C_5’_), 119.7 (C_3’_), 116.4 (C_11_), 67.4 (C_9_), 59.8 (C_8_), 49.3 (C_2_), 48.5 (C_6_), 36.9 (C_3_), 27.3 (C_4_), 22.5 (C_5_), 17.4 ppm (C_7_). MS (ESI): m/z calcd. for (C_19_H_23_ClN_2_O): 330.86, found: 295.34 [M‐Cl]^+^, 34.88 [Cl]^−^, 329.54 [M‐H]^–^. Elemental analysis calcd. (%) for (C_19_H_23_ClN_2_O): C (68.98), H (7.01), N (8.47), found: C (68.51), H (6.97), N (8.98).

### 4.3 Synthesis of Quinine Hydrochloride (**4**)

A 37% aqueous solution of HCl (0.046 mL, 5.5×10^−4^ mol) was diluted with THF (1.0 mL) and added dropwise under stirring to a (–)‐quinine (0.15 g, 4.6×10^−4^ mol) THF (35 mL) solution. A white solid precipitated and after concentrating the solvent to half of the starting volume, the solid was collected in a filter, washed three times with Et_2_O and dried under vacuum at 50 °C for 6 h (95% yield).

${\left[\alpha \right]}_{D}^{22}$ = ‐196.3° (3.0×10^−3^ M, MeOH). ^1^H NMR (400 MHz, CD_3_OD): δ 9.03 (d, *J* = 5.7 Hz, 1H, H_2’_), 8.36 (d, *J* = 5.7 Hz, 1H, H_3’_), 8.23 (d, *J* = 9.3 Hz, 1H, H_8’_), 7.91 (d, *J* = 2.3 Hz, 1H, H_5’_), 7.86 (dd, *J* = 9.3, 2.4 Hz, 1H, H_7’_), 6.55 (s, 1H, H_9_), 5.86–5.77 (m, 1H, H_10_), 5.16 (d, *J* = 17.1 Hz, 1H, H_11A_), 5.06 (d, *J* = 10.4 Hz, 1H, H_11B_), 4.34–4.24 (m, 1H, H_6A_), 4.20 (s, 3H, OCH_3_), 3.73 (pt, *J* = 8.8 Hz, 1H, H_8_), 3.65 (pt, *J* = 7.9 Hz, 1H, H_2A_), 3.38–3.34 (m, 1H, H_6B_), 3.31–3.28 (m, 1H, H_2B_), 2.91–2.82 (m, 1H, H_3_), 2.31–2.21 (m, 2H, H_5A,7A_), 2.15 (d, *J* = 2.7 Hz, 1H, H_4_), 2.06–1.97 (m, 1H, H_5B_), 1.63 ppm (pt, *J* = 8.0 Hz, 1H, H_7B_). ^13^C NMR (101 MHz, CD_3_OD): δ 161.2 (C_6’_), 156.6 (C_10’_), 140.4 (C_2’_), 137.8 (C_10_), 133.7 (C_4’_), 128.0 (C_7’_), 127.8 (C_9’_), 122.8 (C_8’_), 120.1 (C_3’_), 115.9 (C_11_), 102.2 (C_5’_), 66.8 (C_9_), 59.5 (C_8_), 57.1 (OCH_3_), 54.2 (C_2_), 43.8 (C_6_), 37.1 (C_3_), 27.0 (C_4_), 23.7 (C_5_), 17.8 ppm (C_7_). LR‐MS (ESI): m/z calcd. for (C_20_H_25_ClN_2_O_2_): 360.88, found: 295.34 [M‐Cl]^+^. Elemental analysis calcd. (%) for (C_20_H_25_ClN_2_O_2_): C (66.56), H (6.98), N (7.76), found: C (66.91), H (6.57), N (7.56).

### 4.4 General Catalytic Procedure for the Cycloaddition of CO_2_ to Aziridines at Atmospheric Pressure

In a 10.0 mL test tube equipped with a screw cap with a silicon/PTFE septum, cinchonine hydrochloride **3** (0.026 g, 8 × 10^−5^ mol) was dissolved in the desired solvent (0.5 mL) and then 1‐butyl‐2‐phenylaziridine (0.150 mL, 8 × 10^−4^ mol) was added. At this point, CO_2_ was bubbled into the mixture for 5 min, and then a plastic balloon filled with CO_2_ was attached to the top of the flask to maintain a carbon dioxide atmosphere. The reaction was stirred at 30 °C for 16 h, then the CO_2_ balloon was removed, the solvent was evaporated to dryness and the crude analyzed by ^1^H NMR spectroscopy by using 2,4‐dinitrotoluene as the internal standard.

## Conflict of Interest Statement

The authors declare no conflict of interest.

## Supporting information



Supporting Information

## Data Availability

The data that support the findings of this study are available from the corresponding author upon reasonable request.
