# Supplementary material for: A Biocompatible Cinchonine‐Based Catalyst for the CO2 Valorization into Oxazolidin‐2‐ones Under Ambient Conditions
Source: Chemistry. 2025 Mar 26;31(25):e202500473. doi: 10.1002/chem.202500473 (PMC12057589; doi:10.1002/chem.202500473)
Supplement: Supplementary file 1 — Supporting Information [file CHEM-31-e202500473-s001.pdf]

Chemistry – A European Journal

Supporting Information

A Biocompatible Cinchonine-based Catalyst for the CO_2 Valorization into Oxazolidin-2-ones under Ambient Conditions

Lucia Invernizzi,[a] Caterina Damiano[a] and Emma Gallo*[a]

[a] L. Invernizzi, Dr. C. Damiano, Prof. E. Gallo
Department of Chemistry
University of Milan
Via C. Golgi 19, 20133 Milan (Italy)
E-mail: emma.gallo@unimi.it

Table of contents

Synthesis of the catalysts S3

Synthesis of 1,8-diazabicyclo[5.4.0]undec-7-enium chloride (DBUHCl, **2**) S3

Comparison of catalytic systems S3

Optimization of the reaction conditions S4

Study of the reaction dependence on the catalytic loading S4

Study of the reaction dependence on the substrate concentration S4

Study of the reaction dependence on the CO_2 pressure S5

Study of the reaction dependence on the solvent S6

Catalytic test in the absence of CO_2 S6

Calibration curve of 3-butyl-5-phenyloxazolidin-2-one (**6**) in CH_3CN/DMSO=93:7 S7

Study of the reaction dependence on the time S8

Recycling test S9

Recovery of the pre-catalyst S9

Synthesis of oxazolidin-2-ones S9

Synthesis and characterization of (*S*)-1-benzyl-2-octylaziridine (**20**) S13

Mechanistic study S15

NMR spectra of the catalysts S17

NMR spectra of oxazolidin-2-ones S20

NMR spectra of (*R*)-2-chlorodecan-1-ol S30

NMR spectra of (*S*)-1-benzyl-2-octylaziridine (**20**) S31

References S32

Synthesis of the catalysts

Synthesis of 1,8-diazabicyclo[5.4.0]undec-7-enium chloride (DBUHCl, 2)

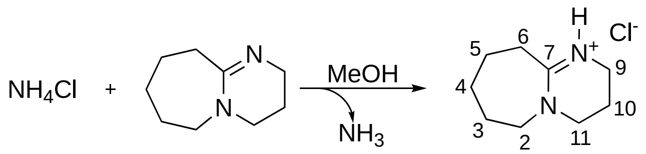

DBU (0.92 g, 6.0 x 10^{-3} mol) was slowly added over 30 minutes to an ammonium chloride (0.33 g, 6.2 x 10^{-3} mol) methanol (4.0 mL) suspension under nitrogen atmosphere. The mixture was stirred at room temperature for 3 hours and then the solvent was evaporated to dryness, affording 1,8-diazabicyclo[5.4.0]undec-7-enium chloride (**2**) as a white glassy solid, which was dried under vacuum at 60 °C for 24 hours (80% yield). Collected data are in accordance with those reported in literature.[1]

^{1}H NMR (400 MHz, $CDCl_3$): δ 11.53 (bs, 1H, NH), 3.55 – 3.46 (m, 6H, $H_{2\text{-}9\text{-}11}$), 3.02 (s, 2H, H_6), 2.05 (p, *J* = 5.8 Hz, 2H, H_{10}), 1.74 ppm (dd, *J* = 15.2, 2.2 Hz, 6H, $H_{3\text{-}4\text{-}5}$). ^{13}C NMR (101 MHz, $CDCl_3$): δ 166.2 (C_7), 54.5 (C_9), 48.8 (C_{11}), 37.9 (C_2), 32.2 (C_6), 29.0 (C_3), 26.8 (C_4), 24.0 (C_5), 19.5 ppm (C_{10}). LR-MS (ESI) m/z calcd. for ($C_9H_{17}N_2Cl$): 188.56, found: 34.88 $[Cl]^-$, 153.08 $[DBUH]^+$.

Comparison of catalytic systems

Method A

In a 3.5 mL glass liner equipped with a screw cap and glass wool, the desired catalyst (8 x 10^{-6} mol) was dissolved in ^{i}PrOH (0.5 mL), then 1-butyl-2-phenylaziridine (0.150 mL, 8 x 10^{-4} mol) was poured into the glass liner. The vessel was transferred into a stainless-steel autoclave and 1.2 MPa of CO_2 pressure were charged at room temperature. The autoclave was placed in a preheated oil bath at 100 °C, stirred for 16 hours and then quenched in an ice bath and slowly vented. The solvent was evaporated to dryness and the crude was analyzed by ^{1}H NMR spectroscopy by using 2,4-dinitrotoluene as the internal standard. Obtained data are reported in the manuscript (Table 1).

Method B

Method A was followed by working at 30 °C. Obtained data are reported in the manuscript (Table 1).

Method C

The desired catalyst (8 x 10^{-6} mol) was dissolved in ^{i}PrOH (0.5 mL) in a 10.0 mL test tube equipped with a screw cap with a silicon/PTFE septum. After adding 1-butyl-2-phenylaziridine (0.150 mL, 8 x 10^{-4} mol), CO_2 was bubbled into the mixture for 5 min and subsequently a plastic balloon filled with CO_2 was attached to the top of the reactor to maintain a carbon dioxide atmosphere. The reaction was stirred at 30 °C for 16 hours, then the CO_2 balloon was removed, the solvent was evaporated to dryness and the crude was analyzed by ^{1}H NMR spectroscopy by using 2,4-dinitrotoluene as the internal standard. Obtained data are reported in the manuscript (Table 1).

Optimization of the reaction conditions

Study of the reaction dependence on the catalytic loading

The desired amount of cinchonine hydrochloride **3** was dissolved in ^{i}PrOH (0.5 mL) in a 10.0 mL test tube equipped with a screw cap with a silicon/PTFE septum. After adding 1-butyl-2-phenylaziridine (0.150 mL, 8 x 10^{-4} mol), CO_2 was bubbled into the mixture for 5 min and subsequently a plastic balloon filled with CO_2 was attached to the top of the reactor to maintain the carbon dioxide atmosphere. The reaction was stirred at 30 °C for 16 hours, then the CO_2 balloon was removed, the solvent was evaporated to dryness and the crude was analyzed by ^{1}H NMR spectroscopy by using 2,4-dinitrotoluene as the internal standard. Obtained data are reported in Table S1.

Table S1. Study of the reaction dependence on the catalytic loading.

Entry	Loading (mol%)	Conversion (%)	Selectivity (%)	Yield (%)	A/B ratio
1	1	20	55	11	95:5
2	5	32	63	20	97:3
3	10	50	74	37	99:1
4[a]	10	57	60	34	97:3
5	15	75	71	57	97:3

[a] Catalyst: quinine hydrochloride (**4**).

Study of the reaction dependence on the substrate concentration

Cinchonine hydrochloride **3** (0.026 g, 8 x 10^{-5} mol) was dissolved in the desired amount of ^{i}PrOH in a 10.0 mL test tube equipped with a screw cap with a silicon/PTFE septum. After adding 1-butyl-2-phenylaziridine (0.150 mL, 8.0 x 10^{-4} mol), CO_2 was bubbled into the mixture for 5 min and subsequently a plastic balloon filled with CO_2 was attached to the top of the reactor to maintain the carbon dioxide atmosphere. The reaction was stirred at 30 °C for 16 hours, then the CO_2 balloon was removed, the solvent was evaporated to dryness and the crude was analyzed by ^{1}H NMR spectroscopy by using 2,4-dinitrotoluene as the internal standard. Obtained data are reported in Table S2.

Table S2. Study of the reaction dependence on the substrate concentration.

Entry	V_{iPrOH} (mL)	[AZI] (M)	Conversion (%)	Selectivity (%)	Yield (%)	A/B ratio
1	1.0	0.8	40	50	20	98:2
2	0.5	1.6	50	74	37	99:1
3	0.25	3.2	46	67	31	98:2
4	0.125	6.4	46	61	28	98:2

Study of the reaction dependence on the CO_2 pressure

In a 3.5 mL glass liner with a screw cap and glass wool, cinchonine hydrochloride **3** (8×10^{-5} mol) was dissolved in ^{i}PrOH (0.5 mL), then 1-butyl-2-phenylaziridine (0.150 mL, 8×10^{-4} mol) was poured into the glass liner. The vessel was transferred into a stainless-steel autoclave and the desired CO_2 pressure was charged at room temperature. The autoclave was placed in a preheated oil bath at 30 °C, stirred for 16 hours and then slowly vented. The solvent was evaporated to dryness and the crude was analyzed by ^{1}H NMR spectroscopy by using 2,4-dinitrotoluene as the internal standard. Obtained data are reported in Table S3.

Table S3. Study of the reaction dependence on the CO_2 pressure.

Entry	p_{CO2} (MPa)	Conversion (%)	Selectivity (%)	Yield (%)	A/B ratio
1	0.1	50	74	37	99:1
2	0.2	90	82	74	97:3
3	0.3	100	80	74	96:4
4	0.6	100	82	82	97:3
5	0.9	100	85	85	99:1
6	1.2	100	90	90	98:2

Study of the reaction dependence on the solvent

Cinchonine hydrochloride **3** (0.026 g, 8 x 10^{-5} mol) was dissolved in the desired solvent (0.5 mL) in a 10.0 mL test tube equipped with a screw cap with a silicon/PTFE septum. After adding 1-butyl-2-phenylaziridine (0.150 mL, 8 x 10^{-4} mol), CO_2 was bubbled into the mixture for 5 min and subsequently a plastic balloon filled with CO_2 was attached to the top of the reactor to maintain the carbon dioxide atmosphere. The reaction was stirred at 30 °C for 16 hours, then the CO_2 balloon was removed, the solvent was evaporated to dryness and the crude was analyzed by ^{1}H NMR spectroscopy by using 2,4-dinitrotoluene as the internal standard. Obtained data are reported in Table S4.

Table S4. Study of the reaction dependence on the solvent.

Entry	Solvent	Conversion (%)	Selectivity (%)	Yield (%)	A/B ratio
1	H_2O	9	81	7	99:1
2	H_2O/AcOEt=1:1	25	92	23	98:2
3	MeOH	32	63	20	95:5
4	EtOH	39	77	30	98:2
5	^{i}PrOH	50	74	37	99:1
6	^{i}PrOH/AcOEt=1:1	45	84	38	97:3
7	^{i}PrOH/MEK[a]=1:1	54	85	45	96:4
8	^{i}PrOH/THF=1:1	51	88	45	97:3
9	^{i}PrOH/2-MeTHF=1:1	54	74	40	98:2
10	^{i}PrOH/CH_3CN=1:1	65	74	48	97:3
11	MEK[a]	59	81	48	97:3
12	CH_3CN	73 (78)[b]	92 (92)[b]	67 (72)[b]	97:3 (98:2)[b]
13	CH_3CN/H_2O=96:4	81	80	65	97:3
14	CH_3CN/MeOH=96:4	81	80	65	96:4
15	CH_3CN/DMSO=96:4	83	100	83	96:4
16	CH_3CN/DMSO=93:7	91	100	91	96:4
17	CH_3CN/DMSO=9:1	100	85	85	96:4

[a] MEK: methyl ethyl ketone. [b] The reaction was run for 24 hours.

Catalytic test in the absence of CO_2

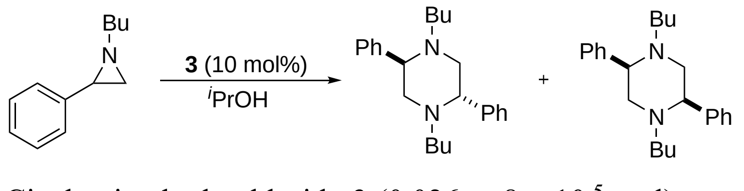

Cinchonine hydrochloride **3** (0.026 g, 8 x 10^{-5} mol) was dissolved in ^{i}PrOH (0.5 mL) in a 10.0 mL test tube equipped with a screw cap with a silicon/PTFE septum, then 1-butyl-2-phenylaziridine (0.150 mL, 8 x 10^{-4} mol) was added and the reaction was stirred at 30 °C for 16 hours. At the end, the

solvent was evaporated to dryness and the crude was analyzed by ^1H NMR spectroscopy by using 2,4-dinitrotoluene as the internal standard. The formation of a mixture of the different isomers of 1,4-dibutyl-2,5-diphenylpiperazine[2] was observed. The obtained mixture was not further investigated.

Calibration curve of 3-butyl-5-phenyloxazolidin-2-one (6) in CH_3CN/DMSO=93:7

The solutions of 3-butyl-5-phenyloxazolidin-2-one (**6**) in CH_3CN/DMSO=93:7 reported in Table S4 were prepared by appropriately diluting a 0.148 M solution. IR spectra were recorded in the range 2100–1600 cm^{-1} by using a CaF_2 cell with an optical path length of 0.5 cm. The calibration curve (Figure S4) was constructed by monitoring the absorbance of the peak at 1751 cm^{-1}, related to C=O stretching. Obtained data are reported in Table S4.

Table S4. Data employed for the calibration curve.

Entry	[OXA] (M)	A
1	1.48×10^{-3}	0.0923
2	2.96×10^{-3}	0.1973
3	5.92×10^{-3}	0.3920
4	7.40×10^{-3}	0.4256
5	1.48×10^{-2}	0.8253

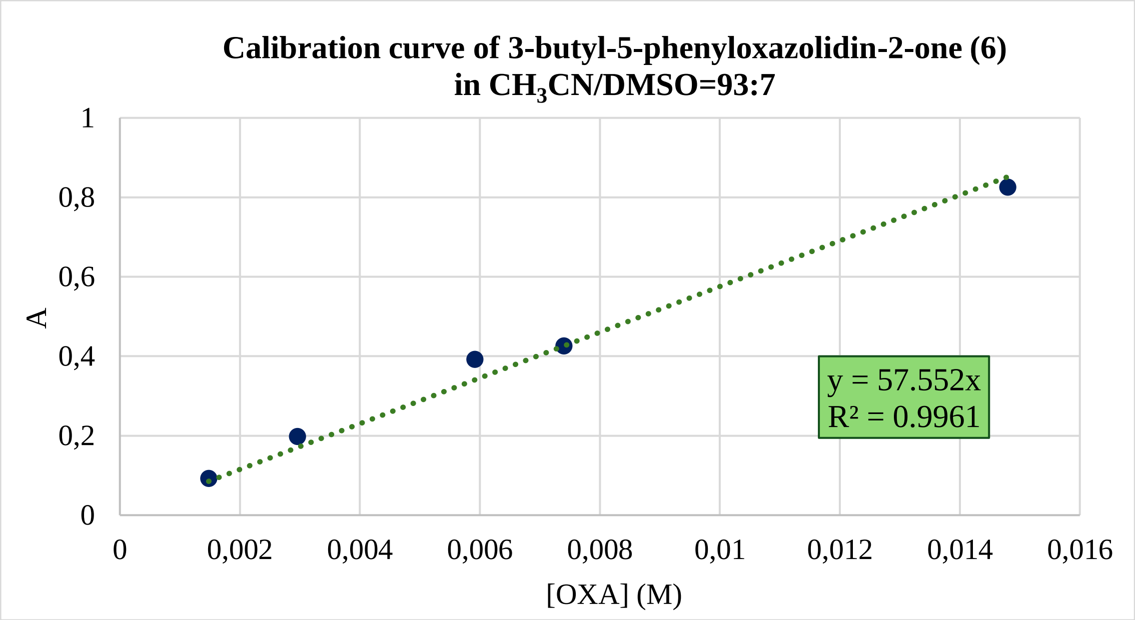

Figure S4. IR calibration curve for 3-butyl-5-phenyloxazolidinone (**6**) in CH_3CN/DMSO=93:7.

According to the Lambert-Beer law, the molar extinction coefficient for 3-butyl-5-phenyloxazolidin-2-one (**6**) in CH_3CN/DMSO=93:7 resulted to be **ε = 115.1 L mol^{-1} cm^{-1}**.

Study of the reaction dependence on the time

In a two-necked round-bottom flask, cinchonine hydrochloride **3** (0.078 g, 2.4 x 10^{-4} mol) was dissolved in CH_3CN/DMSO=93:7 (1.5 mL), then 1-butyl-2-phenylaziridine (0.450 mL, 2.4 x 10^{-3} mol) was added. At this point, a glass cap and a silicon septum were placed on the flask's necks and CO_2 was bubbled into the reaction mixture for 5 min. Subsequently, a plastic balloon filled with CO_2 was attached to the top of the reactor to maintain the carbon dioxide atmosphere. The reaction was stirred at 30 °C and the formation of 3-butyl-5-phenyloxazolidin-2-one (**6**) over time was monitored *via* liquid IR absorption spectroscopy by taking, at regular time intervals, known amounts of the crude, which were analyzed after diluting by a factor of 100. The absorbance of the peak at 1751 cm^{-1} was monitored and the previously calculated ε value was employed to determine the amount of **6** formed at each reaction stage. Obtained data are reported in Table S5 and Figure S5.

Table S5. Study of the reaction dependence on time.

Entry	*t* (min)	[OXA] (M)	Entry	*t* (min)	[OXA] (M)
1	20	0.063	9	300	1.272
2	40	0.145	10	360	1.634
3	60	0.249	11	420	1.638
4	80	0.318	12	450	1.672
5	100	0.394	13	480	1.682
6	120	0.442	14	540	1.690
7	180	0.682	15	600	1.736
8	240	1.029	16	780	1.760

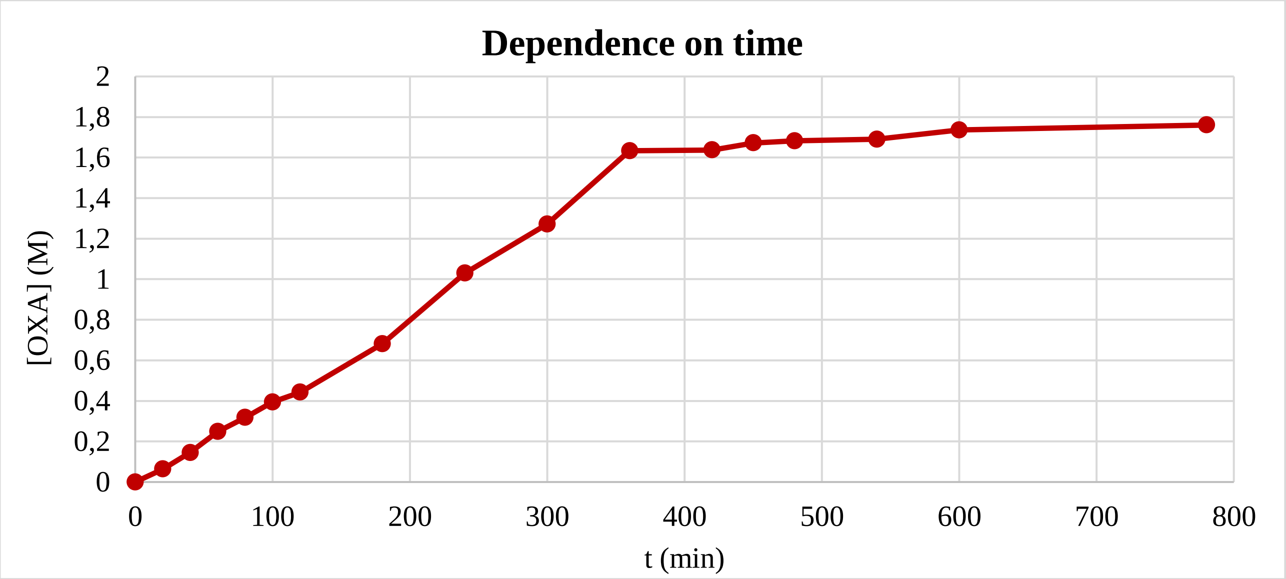

Figure S5. Formation of 3-butyl-5-phenyloxazolidin-2-one (**6**) over time.

Recycling test

Cinchonine hydrochloride **3** (0.026 g, 8 x 10^{-5} mol) was dissolved in CH_3CN/DMSO=93:7 (0.5 mL) in a 10.0 mL test tube equipped with a screw cap with a silicon/PTFE septum. After adding 1-butyl-2-phenylaziridine (0.150 mL, 8 x 10^{-4} mol), CO_2 was bubbled into the reaction mixture for 5 min and subsequently a plastic balloon filled with CO_2 was attached to the top of the reactor to maintain the carbon dioxide atmosphere. The reaction was stirred at 30 °C for 16 hours, then the CO_2 balloon was removed and the same amount of substrate was added again. A new CO_2 balloon was placed on the reactor and the reaction was carried out for further 16 hours. The above-described procedure was repeated for three consecutive times, then the solvent was evaporated to dryness and the crude was analyzed by ^{1}H NMR spectroscopy by using 2,4-dinitrotoluene as the internal standard. The desired product (**6**) was obtained in 84% of global yield (100% selectivity) and A/B = 95:5.

Recovery of the pre-catalyst

Cinchonine hydrochloride **3** (0.026 g, 8 x 10^{-5} mol) was dissolved in CH_3CN/DMSO=93:7 (0.5 mL) in a 10.0 mL test tube equipped with a screw cap with a silicon/PTFE septum. After adding 1-butyl-2-phenylaziridine (0.150 mL, 8 x 10^{-4} mol), CO_2 was bubbled into the mixture for 5 min and subsequently a plastic balloon filled with CO_2 was attached to the top of the reactor to maintain the carbon dioxide atmosphere. The reaction was stirred at 30 °C for 16 hours, then the CO_2 balloon was removed and CH_2Cl_2 (5 mL) and H_2O (5 mL) were added. The organic layer was washed three times with H_2O, then the aqueous phases were combined and placed in an ice-water bath. While stirring, a 0.1 M aqueous solution of NaOH (1.5 mL) was added in small portions and the precipitation of a white solid was observed. The mixture was kept under stirring for further 30 min, then the white precipitate was filtered, washed three times with H_2O and dried under vacuum at 50 °C for 5 hours. The recovered solid (0.030 g) was analyzed *via* ^{1}H NMR spectroscopy and its spectrum in $CDCl_3$ was perfectly superimposable with the one of the commercially available (+)-cinchonine.

Synthesis of oxazolidin-2-ones

Synthesis of 3-butyl-5-phenyloxazolidin-2-one (6)

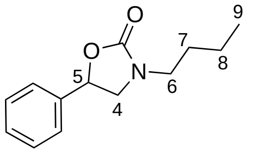

The general catalytic procedure was followed by using 1-butyl-2-phenylaziridine (0.150 mL) as the substrate to obtain a yellowish oil as the product (91% yield, **A**/**B** 96:4 by using CH_3CN/DMSO=93:7 as the solvent; 37% yield, **A**/**B** 99:1 by using ^{i}PrOH as the solvent). Collected data are in accordance with those reported in literature.[6]

^{1}H NMR (300 MHz, $CDCl_3$) δ 7.45 – 7.28 (m, 5H, H_{Ar}), 5.50 (pt, J = 8.0 Hz, 1H, H_5), 3.93 (pt, J = 8.8 Hz, 1H, H_{4A}), 3.44 (pt, J = 8.0 Hz, 1H, H_{4B}), 3.36 – 3.23 (m, 2H, H_6), 1.61 – 1.51 (m, 2H, H_7), 1.43 – 1.31 (m, 2H, H_8), 0.96 ppm (t, J = 6.8 Hz, 3H, H_9). ^{13}C NMR (75 MHz, $CDCl_3$) δ 139.32 (C_{Ar}), 129.30 (C_{Ar}), 129.16 (C_{Ar}), 127.41 (C_{Ar}), 125.90 (C_{Ar}), 74.71 (C_4), 52.58 (C_6), 44.32 (C_5), 29.81 (C_7), 20.24 (C_8), 14.10 ppm (C_9). LR-MS (ESI) m/z calcd. for ($C_{13}H_{17}NO_2$): 219.13, found 242.22 $[M+Na]^+$.

Synthesis of 3-propyl-5-phenyloxazolidin-2-one (7)

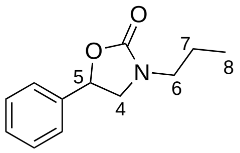

The general catalytic procedure was followed by using 1-propyl-2-phenylaziridine (0.138 mL) as the substrate to obtain a yellowish oil as the product (85% yield, **A**/**B** 94:6 by using CH_3CN/DMSO=93:7 as the solvent; 51% yield, **A**/**B** 98:2 by using ^{i}PrOH as the solvent). Collected data are in accordance with those reported in literature.[5]

^{1}H NMR (300 MHz, $CDCl_3$) δ 7.40 – 7.27 (m, 5H, H_{Ar}), 5.47 (pt, J = 8.0 Hz, 1H, H_5), 3.90 (pt, J = 8.8 Hz, 1H, H_{4A}), 3.41 (pt, J = 8.0 Hz, 1H, H_{4B}), 3.37 – 3.19 (m, 2H, H_6), 1.60 – 1.55 (m, 2H, H_7), 0.92 ppm (t, J = 6.8 Hz, 3H, H_8). ^{13}C NMR (75 MHz, $CDCl_3$) δ 157.97 (C=O), 138.95 (C_{Ar}), 128.87 (C_{Ar}), 128.73 (C_{Ar}), 126.98 (C_{Ar}), 125.50 (C_{Ar}), 74.31 (C_4), 52.12 (C_6), 45.79 (C_5), 20.64 (C_7), 11.07 ppm (C_8). LR-MS (ESI) m/z calcd. for ($C_{12}H_{15}NO_2$): 205.11, found 206.04 $[M+H]^+$.

Synthesis of 3-isobutyl-5-phenyloxazolidin-2-one (8)

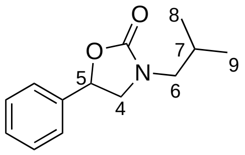

The general catalytic procedure was followed by using 1-isobutyl-2-phenylaziridine (0.153 mL) as the substrate to obtain a yellowish oil as the product (78% yield, **A**/**B** 99:1 by using CH_3CN/DMSO=93:7 as the solvent; 49% yield, **A**/**B** 100:0 by using ^{i}PrOH as the solvent). Collected data are in accordance with those reported in literature.[6]

^{1}H NMR (300 MHz, $CDCl_3$) δ 7.41 – 7.24 (m, 5H, H_{Ar}), 5.47 (pt, J = 8.4 Hz, 1H, H_5), 3.90 (pt, J = 8.8 Hz, 1H, H_{4A}), 3.42 (dd, J = 8.7, 7.4 Hz, 1H, H_{4B}), 3.16 – 3.01 (m, 2H, H_6) 1.92 – 1.80 (m, 1H, H_7), 0.92 (pt, J = 6.8 Hz, 6H, $H_{8\text{-}9}$). ^{13}C NMR (75 MHz, $CDCl_3$) δ 158.62 (C=O), 139.37 (C_{Ar}), 129.27 (C_{Ar}), 129.13 (C_{Ar}), 127.39 (C_{Ar}), 125.88 (C_{Ar}), 74.69 (C_4), 53.17 (C_6), 52.15 (C_5), 27.21 (C_7), 20.31 (C_8), 20.28 ppm (C_9). LR-MS (ESI) m/z calcd. for ($C_{13}H_{17}NO_2$): 219.13, found 242.18 $[M+Na]^+$.

Synthesis of 3-secbutyl-5-phenyloxazolidin-2-one (9)

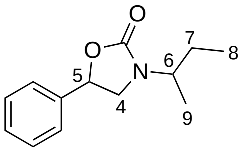

The general catalytic procedure was followed by using 1-secbutyl-2-phenylaziridine (0.153 mL) as the substrate to obtain a yellowish oil as the product (21% yield, **A**/**B** 100:0 by using CH_3CN/DMSO=93:7 as the solvent; 14% yield, **A**/**B** 98:2 by using ^{i}PrOH as the solvent). Collected data are in accordance with those reported in literature.[7]

^{1}H NMR (300 MHz, $CDCl_3$): δ 7.44 – 7.35 (m, 5H, H_{Ar}), 5.51 (pt, J = 7.8 Hz, 1H, H_5), 3.98 – 3.86 (m, 2H, $H_{4A\text{-}6}$), 3.32 (pt, J = 8.7 Hz, 1H, H_{4B}), 1.53 – 1.45 (m, 2H, H_7), 1.21 (d, J = 6.8 Hz, 3H, H_9), 0.89 ppm (t, J = 7.4 Hz, 3H, H_8). ^{13}C NMR (75 MHz, $CDCl_3$) δ 157.61 (C=O), 139.30 (C_{Ar}), 128.91 (C_{Ar}), 128.71 (C_{Ar}), 125.50 (C_{Ar}), 74.42 (C_6), 50.62 (C_5), 47.41 (C_4), 27.01 (C_7), 18.01 (C_9), 10.88 ppm (C_8). LR-MS (ESI) m/z calcd. for ($C_{13}H_{17}NO_2$) 219.13, found 242.26 $[M+Na]^+$.

Synthesis of 3-isopentyl-5-phenyloxazolidin-2-one (10)

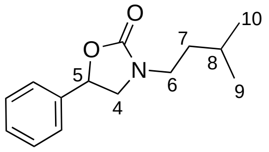

The general catalytic procedure was followed by using 1-isopentyl-2-phenylaziridine (0.166 mL) as the substrate to obtain a yellowish oil as the product (70% yield, **A**/**B** 96:4 by using CH_3CN/DMSO=93:7 as the solvent; 51% yield, **A**/**B** 97:3 by using ^{i}PrOH as the solvent). Collected data are in accordance with those reported in literature.[6]

^{1}H NMR (300 MHz, $CDCl_3$) δ 7.45 – 7.134 (m, 5H, H_{Ar}), 5.50 (pt, J = 8.1 Hz, 1H, H_5), 3.93 (pt, J = 8.8 Hz, 1H, H_{4A}), 3.46 – 3.39 (m, 1H, H_{4B}), 3.38 – 3.25 (m, 2H, H_6), 1.70 – 1.56 (m, 1H, H_8), 1.50 – 1.42 (m, 2H, H_7), 0.97 – 0.94 ppm (m, 6H, $H_{9\text{-}10}$). ^{13}C NMR (75 MHz, $CDCl_3$) δ 129.30 (C_{Ar}), 129.16 (C_{Ar}), 125.90 (C_{Ar}), 74.71 (C_4), 52.53 (C_6), 43.01 (C_5), 36.51 (C_7), 26.15 (C_8), 22.86 (C_9), 22.80 ppm (C_{10}). LR-MS (ESI) m/z calcd. for ($C_{14}H_{19}NO_2$): 233.14, found 234.12 $[M+H]^+$.

Synthesis of 3-hexyl-5-phenyloxazolidin-2-one (11)

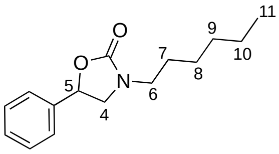

The general catalytic procedure was followed by using 1-hexyl-2-phenylaziridine (0.168 mL) as the substrate to obtain a yellowish oil as the product (81% yield, **A**/**B** 95:5 by using CH_3CN/DMSO=93:7 as the solvent; 48% yield, **A**/**B** 97:3 by using ^{i}PrOH as the solvent). Collected data are in accordance with those reported in literature.[5]

^{1}H NMR (300 MHz, $CDCl_3$) δ 7.38 – 7.24 (m, 5H, H_{Ar}), 5.43 (pt, J = 8.8 Hz, 1H, H_5), 3.88 (pt, J = 8.8 Hz, 1H, H_{4A}), 3.38 (pt, J = 8.7 Hz, 1H, H_{4B}), 3.32 – 3.19 (m, 2H, H_6), 1.53 – 1.50 (m, 2H, H_7), 1.30 – 1.24 (m, 6H, $H_{8,9,10}$), 0.86 ppm (t, J = 6.2 Hz, 3H, H_{11}). ^{13}C NMR (101 MHz, $CDCl_3$) δ 157.86 (C=O), 138.98 (C_{Ar}), 129.66 (C_{Ar}), 128.06 (C_{Ar}), 124.71 (C_{Ar}), 75.03 (C_4), 73.50 (C_6), 52.09 (C_5), 44.14 (C_7), 27.26 (C_8), 26.23 (C_9), 14.55 (C_{10}), 13.31 ppm (C_{11}). LR-MS (ESI) m/z calcd. for ($C_{15}H_{21}NO_2$): 247.34, found 248.17 $[M+H]^+$.

Synthesis of 3-cyclopentyl-5-phenyloxazolidin-2-one (12)

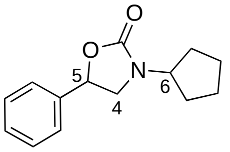

The general catalytic procedure was followed by using 1-cyclopentyl-2-phenylaziridine (0.153 mL) as the substrate to obtain a yellowish oil as the product (26% yield, **A**/**B** 100:0 by using CH_3CN/DMSO=93:7 as the solvent; 18% yield, **A**/**B** 99:1 by using ^{i}PrOH as the solvent). Collected data are in accordance with those reported in literature.[6]

^{1}H NMR (300 MHz, $CDCl_3$) δ 7.45 – 7.30 (m, 5H, H_{Ar}), 5.50 (pt, J = 8.8 Hz, 1H, H_5), 4.37 – 4.29 (m, 1H, H_{4A}), 3.90 (pt, J = 8.7 Hz, 1H, H_{4B}), 3.42 (pt, J = 8.2 Hz, 1H, H_6), 2.00 – 1.88 (m, 3H, $H_{cyclopentyl}$), 1.70 –1.52 ppm (m, 5H, $H_{cyclopentyl}$). ^{13}C NMR (75 MHz, $CDCl_3$) δ 129.30 (C_{Ar}), 129.15 (C_{Ar}), 126.82 (C_{Ar}), 125.89 (C_{Ar}), 74.85 (C_4), 55.06 (C_6), 48.98 (C_5), 29.56 ($C_{cyclopentyl}$), 29.03

($C_{cyclopentyl}$), 24.31 ppm ($C_{cyclopentyl}$). LR-MS (ESI) m/z calcd. for ($C_{14}H_{17}NO_2$): 231.29, found 232.17 $[M+H]^+$.

Synthesis of 3-cyclohexyl-5-phenyloxazolidin-2-one (13)

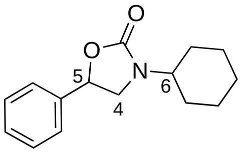

The general catalytic procedure was followed by using 1-cyclohexyl-2-phenylaziridine (0.163 mL) as the substrate to obtain a yellowish oil as the product (21% yield, **A**/**B** 100:0 by using CH_3CN/DMSO=93:7 as the solvent; 11% yield, **A**/**B** n.d. by using ^{i}PrOH as the solvent). Collected data are in accordance with those reported in literature.[6]

^{1}H NMR (300 MHz, $CDCl_3$) δ 7.43 – 7.31 (m, 5H, H_{Ar}), 5.48 (pt, J = 8.8 Hz, 1H, H_6), 3.90 (pt, J = 8.8 Hz, 1H, H_{4B}), 3.82 – 3.71 (m, 1H, H_5), 3.42 (pt, J = 8.7 Hz, 1H, H_{4A}), 1.90 – 1.07 ppm (m, 11H, $H_{cyclohexyl}$). ^{13}C NMR (75 MHz, $CDCl_3$) δ 129.29 (C_{Ar}), 129.12 (C_{Ar}), 128.66 (C_{Ar}), 125.88 (C_{Ar}), 74.98 (C_4), 52.97 (C_6), 48.73 (C_5), 30.98 ($C_{cyclohexyl}$), 30.51 ($C_{cyclohexyl}$), 26.63 ($C_{cyclohexyl}$), 25.51 ppm ($C_{cyclohexyl}$). LR-MS (ESI) m/z calcd. for ($C_{15}H_{19}NO_2$): 245.32, found 268.21 $[M+Na]^+$.

Synthesis of 3-cyclohexanemethyl-5-phenyloxazolidin-2-one (14)

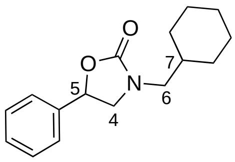

The general catalytic procedure was followed by using 1-cyclohexanemethyl-2-phenylaziridine (0.187 mL) as the substrate to obtain a yellowish oil as the product (74% yield, **A**/**B** 98:2 by using CH_3CN/DMSO=93:7 as the solvent; 62% yield, **A**/**B** 99:1 by using ^{i}PrOH as the solvent). Collected data are in accordance with those reported in literature.[7]

^{1}H NMR (300 MHz, $CDCl_3$) δ 7.39 – 7.31 (m, 5H, H_{Ar}), 5.46 (pt, J = 8.2 Hz, 1H, H_5), 3.89 (pt, J = 8.8 Hz, 1H, H_{4A}), 3.40 (pt, J = 8.7 Hz, 1H, H_{4B}), 3.19 – 3.03 (m, 2H, H_6), 1.72 – 1.63 (m, 6H, $H_{cyclohexyl}$), 1.24 – 1.01 (m, 3H, $H_{cyclohexyl}$), 0.97 – 0.88 ppm (m, 2H, $H_{cyclohexyl}$). ^{13}C NMR (75 MHz, $CDCl_3$) δ 158.57 (C=O), 139.29 (C_{Ar}), 129.22 (C_{Ar}), 129.07 (C_{Ar}), 125.83 (C_{Ar}), 74.67 (C_4), 53.36 (C_6), 50.91 (C_5), 36.41 (C_7), 30.96 ($C_{cyclohexyl}$), 26.66 ($C_{cyclohexyl}$), 26.01 ppm ($C_{cyclohexyl}$). LR-MS (ESI) m/z calcd. for ($C_{16}H_{21}NO_2$): 259.16, found 282.28 $[M+Na]^+$.

Synthesis of 3-benzyl-5-phenyloxazolidin-2-one (15)

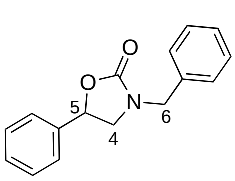

The general catalytic procedure was followed by using 1-benzyl-2-phenylaziridine (0.159 mL) as the substrate to obtain a yellowish oil as the product (80% yield, **A**/**B** 100:0 by using CH_3CN/DMSO=93:7 as the solvent; 53% yield, **A**/**B** 100:0 by using ^{i}PrOH as the solvent). Collected data are in accordance with those reported in literature.[6]

^{1}H NMR (300 MHz, $CDCl_3$) δ 7.41 – 7.29 (m, 10H, H_{Ar}), 5.49 (pt, *J* = 8.5 Hz, 1H, H_5), 4.58 (d, *J* = 15.0 Hz, 1H, H_{6A}), 4.43 (d, *J* = 15.0 Hz, 1H, H_{6B}), 3.79 (pt, *J* = 8.7 Hz, 1H, H_{4A}), 3.33 ppm (pt, *J* = 8.8 Hz, 1H, H_{4B}). ^{13}C NMR (75 MHz, $CDCl_3$) δ 146.73 (C=O), 139.03 (C_{Ar}), 136.08 (C_{Ar}), 129.29 (C_{Ar}), 129.21 (C_{Ar}), 128.58 (C_{Ar}), 128.45 (C_{Ar}), 125.92 (C_{Ar}), 74.93 (C_4), 51.96 (C_6), 48.83 ppm (C_5). LR-MS (ESI) m/z calcd. for ($C_{16}H_{15}NO_2$): 253.11, found 276.27 $[M+Na]^+$.

Synthesis and characterization of (*S*)-1-benzyl-2-octylaziridine (20)

The compound was obtained by slightly modifying a synthetic strategy reported for similar molecules.[3]

Synthesis of (*R*)-2-chlorodecan-1-ol

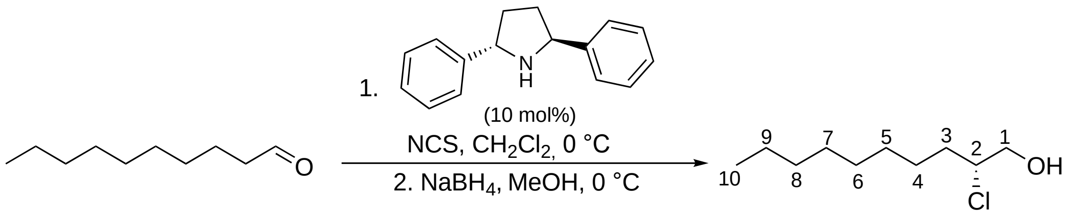

Decanal (0.21 g, 1.3 x 10^{-3} mol) was dissolved in CH_2Cl_2 (3.8 mL) and the solution was cooled to 0 °C, then (2*S*,5*S*)-2,5-diphenylpyrrolidine (0.030 g, 1.3 x 10^{-4} mol) and *N*-chlorosuccinimide (0.23 g, 1.8 x 10^{-3} mol) were added under stirring. The reaction mixture was stirred overnight at 0 °C, then it was warmed to room temperature and analyzed *via* TLC (eluant: *n*-Hexane/AcOEt = 8:2, visualized by using Komarovsky's reagent),[4] observing the complete consumption of the starting material. At this point, the reaction was diluted with MeOH (3.8 mL) and cooled to 0 °C, then $NaBH_4$ (0.25 g, 6.7 x 10^{-3} mol) was added in small portions under stirring. The reaction was stirred for 1 hour at 0 °C and then warmed to room temperature. After that, H_2O (20.0 mL) was added and the mixture was extracted with AcOEt (3 x 20.0 mL). The organic fractions were combined, washed with brine (20.0 mL) and dried over Na_2SO_4. Finally, the solvent was removed under reduced pressure, the crude was purified by flash chromatography (silica gel, gradient elution from *n*-hexane to *n*-hexane/AcOEt = 95:5) and the desired product was obtained as a pale-yellow oil (71% yield).

$[\alpha]_D^{22}$ = +16.5° (9.7 x 10^{-3} M, $CHCl_3$). ^{1}H NMR (400 MHz, $CDCl_3$): δ 4.08 – 4.02 (m, 1H, H_2), 3.84 – 3.78 (m, 1H, H_{1A}), 3.71 – 3.65 (m, 1H, H_{1B}), 1.99 (pt, *J* = 6.8 Hz, 1H, OH), 1.82 – 1.72 (m, 2H, H_4), 1.58 – 1.50 (m, 1H, H_{3A}), 1.47 – 1.41 (m, 1H, H_{3B}), 1.42 – 1.26 (m, 10H, H_{5-9}), 0.91 ppm (t, *J* = 6.5 Hz, 3H, H_{10}). ^{13}C NMR (101 MHz, $CDCl_3$): δ 67.05 (C_1), 65.48 (C_2), 34.27 (C_3), 31.82 (C_4), 29.37 (C_5), 29.18 (C_6), 29.10 (C_7), 26.33 (C_8), 22.63 (C_9), 14.07 ppm (C_{10}). LR-MS (ESI): m/z calcd. for ($C_{10}H_{21}ClO$): 192.73, found: 194.17 $[M+H]^+$. Elemental analysis calcd. (%) for ($C_{10}H_{21}ClO$): C (62.32), H (10.98), N (0.0), found: C (61.31), H (10.96), N (0.0).

Synthesis of (*S*)-1-benzyl-2-octylaziridine (20)

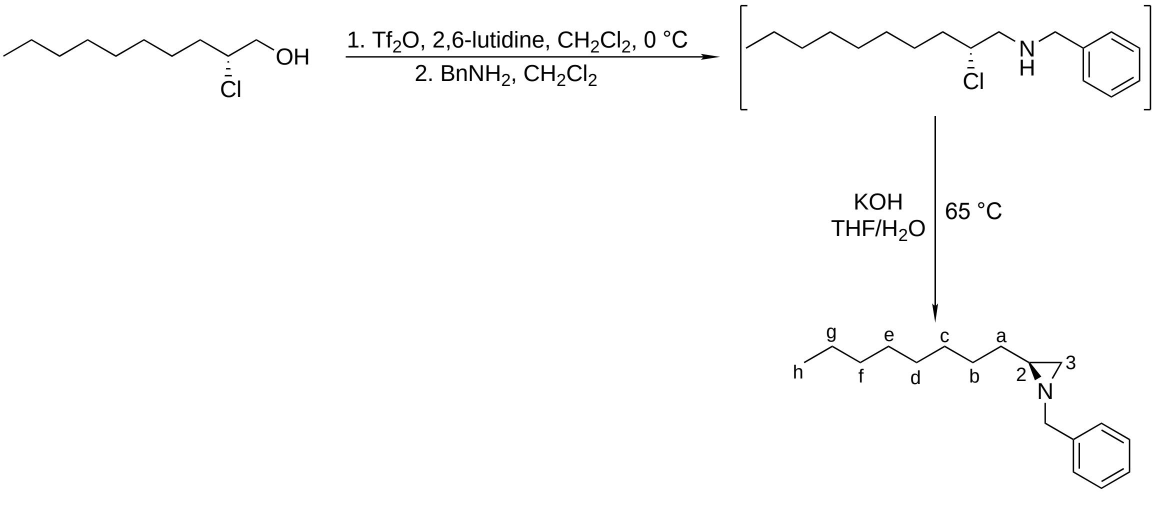

Step 1

(*R*)-2-chlorodecan-1-ol (0.30 g, 1.55 x 10^{-3} mol) and 2,6-lutidine (0.90 g, 8.45 x 10^{-3} mol) were dissolved in CH_2Cl_2 (8.5 mL). The solution was cooled to 0 °C and a solution of Tf_2O (0.95 g, 3.38 x 10^{-3} mol) in CH_2Cl_2 (3.5 mL) was added dropwise over the course of 15 min under stirring. Then, the reaction mixture was kept under stirring at 0 °C for 1.0 h and the obtained solution was employed in *Step 2* without further purification.

Step 2

Benzylamine (1.80 g, 1.7 x 10^{-2} mol) was dissolved in CH_2Cl_2 (3.8 mL) cooled to 0 °C and kept under stirring. The triflate solution prepared in *Step 1* was transferred into a dropping funnel and added dropwise to the amine solution, maintaining the temperature at 0 °C. The reaction mixture was then stirred at 0 °C for 10 min and at room temperature overnight. At this point, the reaction was quenched with a saturated aqueous solution of $NaHCO_3$ (50.0 mL), diluted with CH_2Cl_2 (25.0 mL) and extracted three times with CH_2Cl_2 (50.0 mL). The organic layers were combined, washed twice with brine (100.0 mL) and dried over Na_2SO_4. The solid was filtered off, the solvent was removed under reduced pressure and the obtained crude amine was directly used in the subsequent step without further purifications.

Step 3

The crude amine obtained in *Step 2* (0.44 g, 1.55 x 10^{-3} mol) was dissolved in a 1:1 mixture of THF and H_2O (20.0 mL), then KOH (0.56 g, 1.0 x 10^{-2} mol) was added and the mixture was stirred overnight at 65 °C. The reaction mixture was cooled to room temperature and extracted three times with AcOEt (25.0 mL). The organic layers were combined, dried over Na_2SO_4 and filtered. The resulting solution was evaporated to dryness under reduced pressure and the crude purified by flash chromatography (silica gel, gradient elution from *n*-hexane to *n*-hexane/AcOEt = 99:1; 0.5% of triethylamine was added in order to deactivate the silica). The desired product was obtained as a pale-yellow oil (60% yield).

$[\alpha]_D^{22}$ = -9.3° (5.7 x 10^{-3} M, $CHCl_3$). ^1H NMR (400 MHz, $CDCl_3$): δ 7.38 – 7.35 (m, 4H, H_{Ar}), 7.30 – 7.25 (m, 1H, H_{Ar}), 3.52 (d, J = 13.2 Hz, 1H, H_{benzyl}), 3.34 (d, J = 13.2 Hz, 1H, H_{benzyl}), 1.63 (d, J = 3.2 Hz, 1H, H_{3A}), 1.49 – 1.44 (m, 1H, H_2), 1.43 – 1.38 (m, 3H, $H_{3B,a}$), 1.36 – 1.24 (m, 12H, H_{b-g}), 0.90 ppm (t, J = 6.9 Hz, 3H, H_h). ^{13}C NMR (101 MHz, $CDCl_3$): δ 139.5 (C_{Ar}), 128.3 (C_{Ar}), 128.1 (C_{Ar}), 126.9 (C_{Ar}), 65.0 (C_{benzyl}), 39.8 (C_2), 34.1 (C_3), 33.0 (C_a), 31.9 (C_b), 29.6 (C_c), 29.4 (C_d), 29.2

(C_e), 27.4 (C_f), 22.7 (C_g), 14.1 ppm (C_h). LR-MS (ESI): m/z calcd. for ($C_{17}H_{27}N$): 245.41, found: 246.20 $[M+H]^+$. Elemental analysis calcd. (%) for ($C_{17}H_{27}N$): C (83.20), H (11.09), N (5.71), found: C (82.69), H (11.61), N (5.70).

Mechanistic study

Cinchonine hydrochloride **3** (0.040 g, 1.21 x 10^{-4} mol) was dissolved in CD_3OD (0.75 mL) in an NMR tube equipped with a screw cap and the ^1H NMR spectrum was acquired (Figure S6), then 1-butyl-2-phenylaziridine (0.227 mL, 1.21 x 10^{-3} mol) was added and the mixture was again analyzed *via* ^1H NMR spectroscopy (Figure S7). The tube was saturated with CO_2, sealed and kept at 30 °C for 16 hours. After this time, the ^1H NMR spectrum was registered, and the presence of 3-butyl-5-phenyloxazolidin-2-one (**6**) was observed (Figure S8).

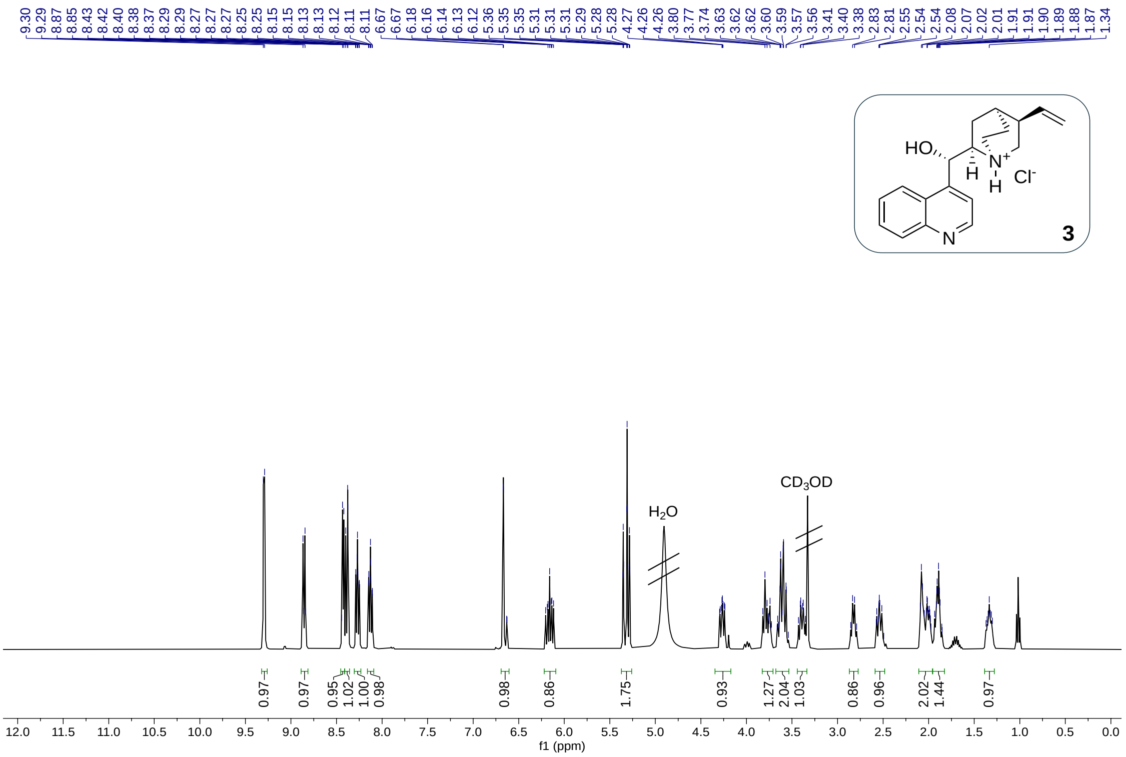

Figure S6. ^1H NMR spectrum of cinchonine hydrochloride (**3**) in CD_3OD.

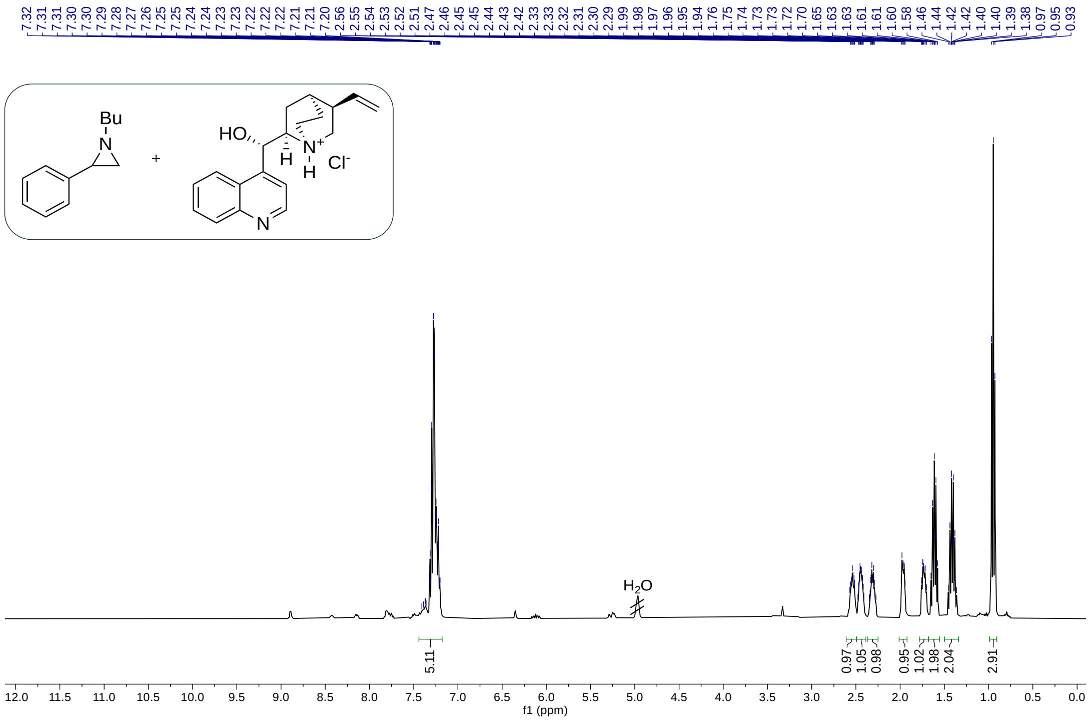

Figure S7. ^{1}H NMR spectrum of cinchonine hydrochloride (**3**) after addition of 1-butyl-2-phenylaziridine.

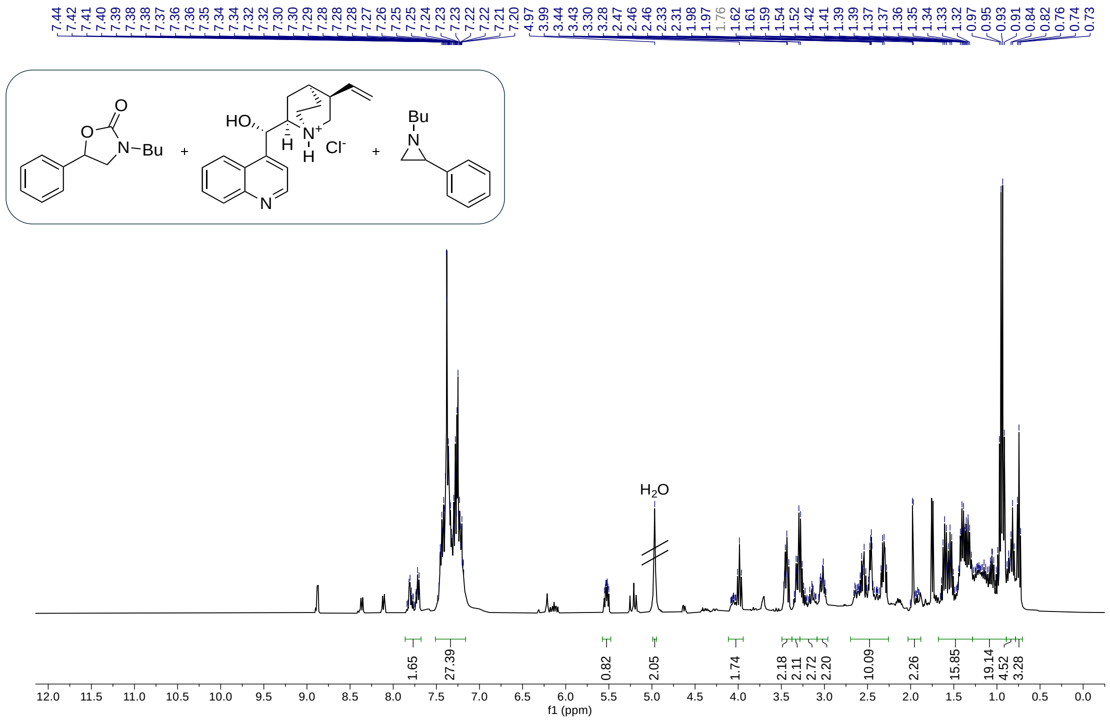

Figure S8. ^{1}H NMR spectrum of the reaction mixture after 16 hours at 30 °C under CO_2 atmosphere.

NMR spectra of the catalysts

NMR spectra of DBUHCl (2)

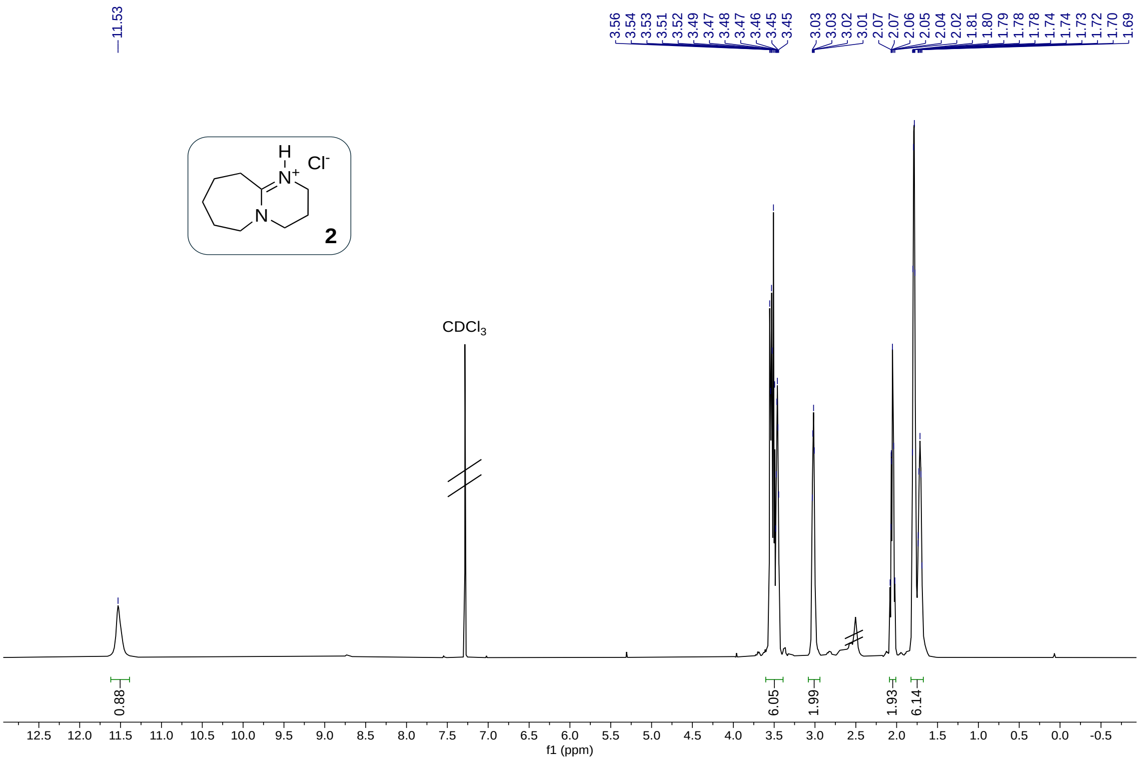

Figure S9. ^{1}H NMR spectrum of DBUHCl (**2**) in $CDCl_3$.

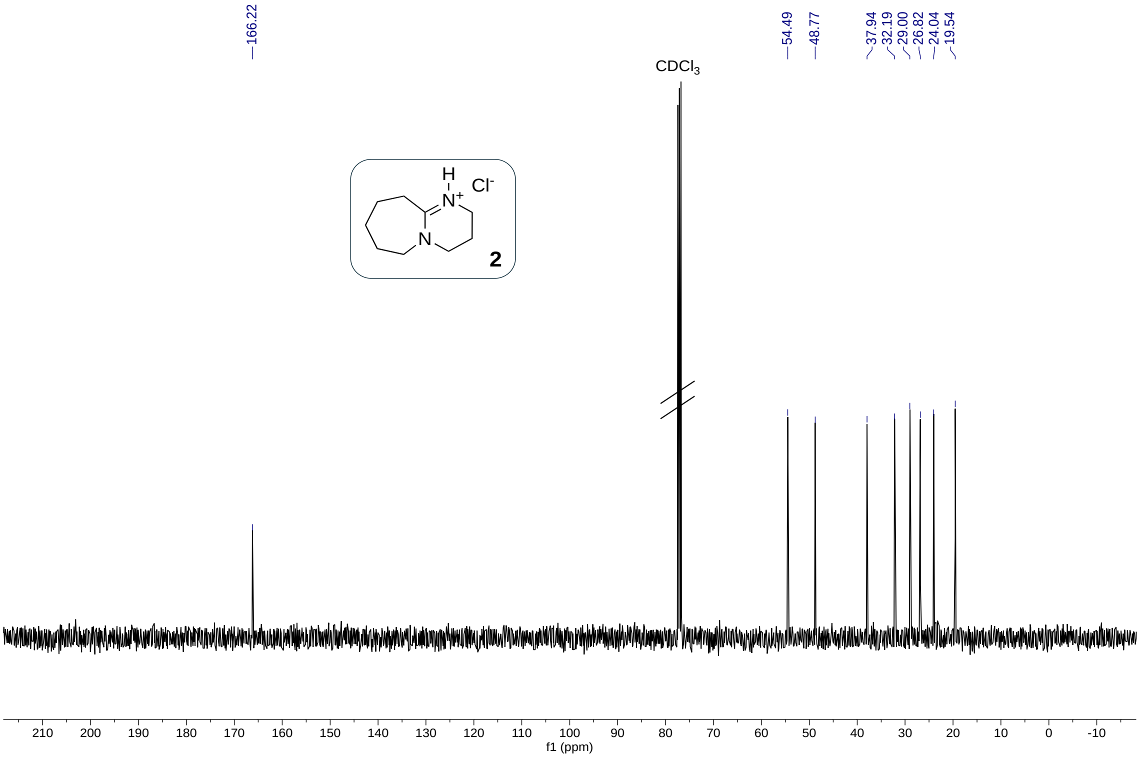

Figure S10. ^{13}C NMR spectrum of DBUHCl (**2**) in $CDCl_3$.

NMR spectra of cinchonine hydrochloride (3)

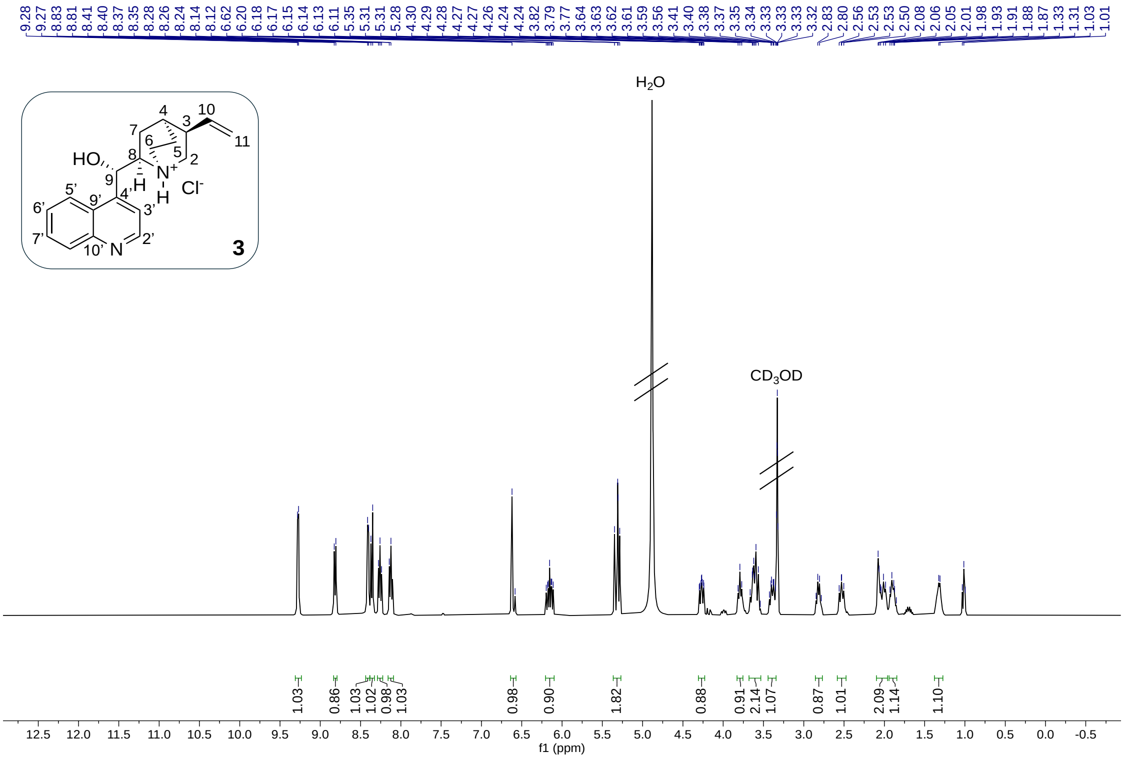

Figure S11. ^{1}H NMR spectrum of cinchonine hydrochloride (**3**) in CD_3OD.

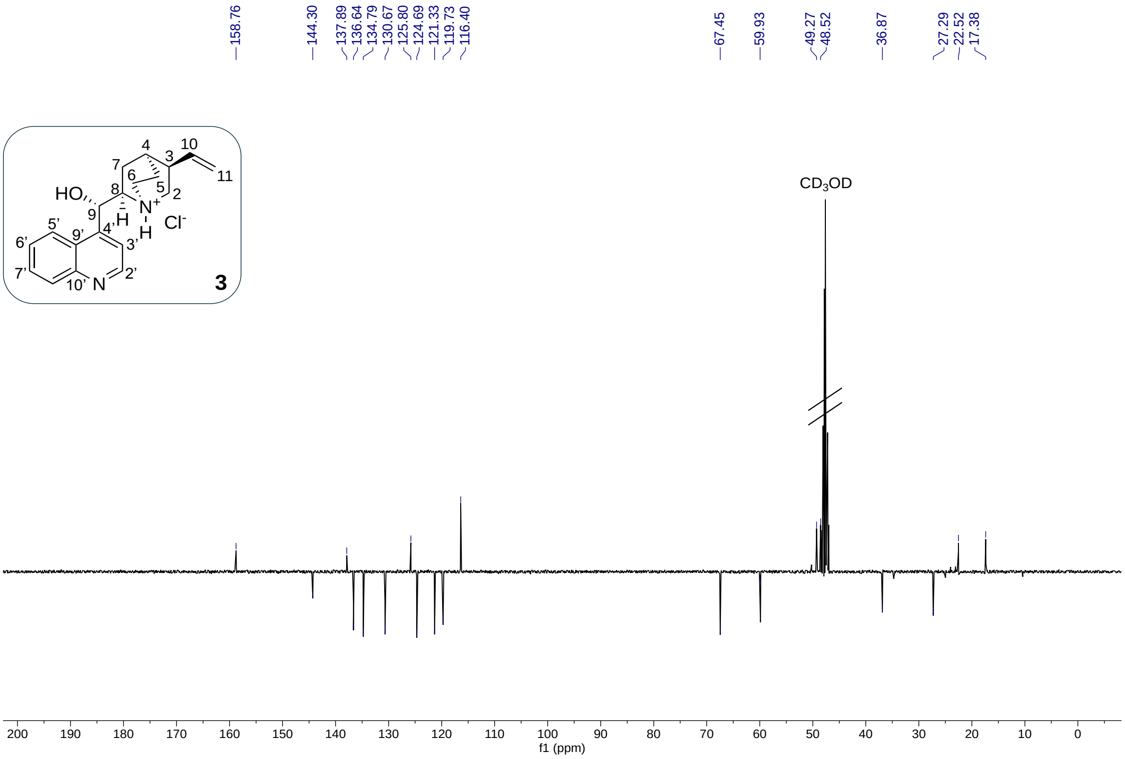

Figure S12. ^{13}C NMR spectrum of cinchonine hydrochloride (**3**) in CD_3OD.

NMR spectra of quinine hydrochloride (4)

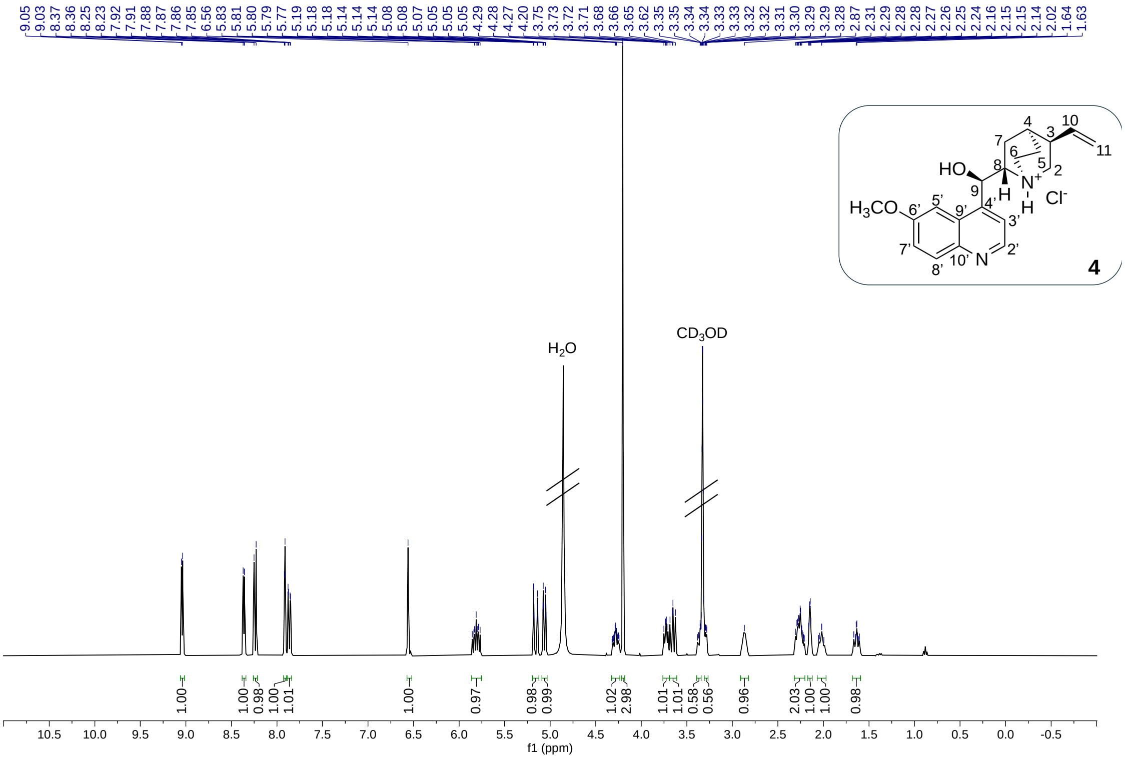

Figure S13. ^{1}H NMR spectrum of quinine hydrochloride (**4**) in CD_3OD.

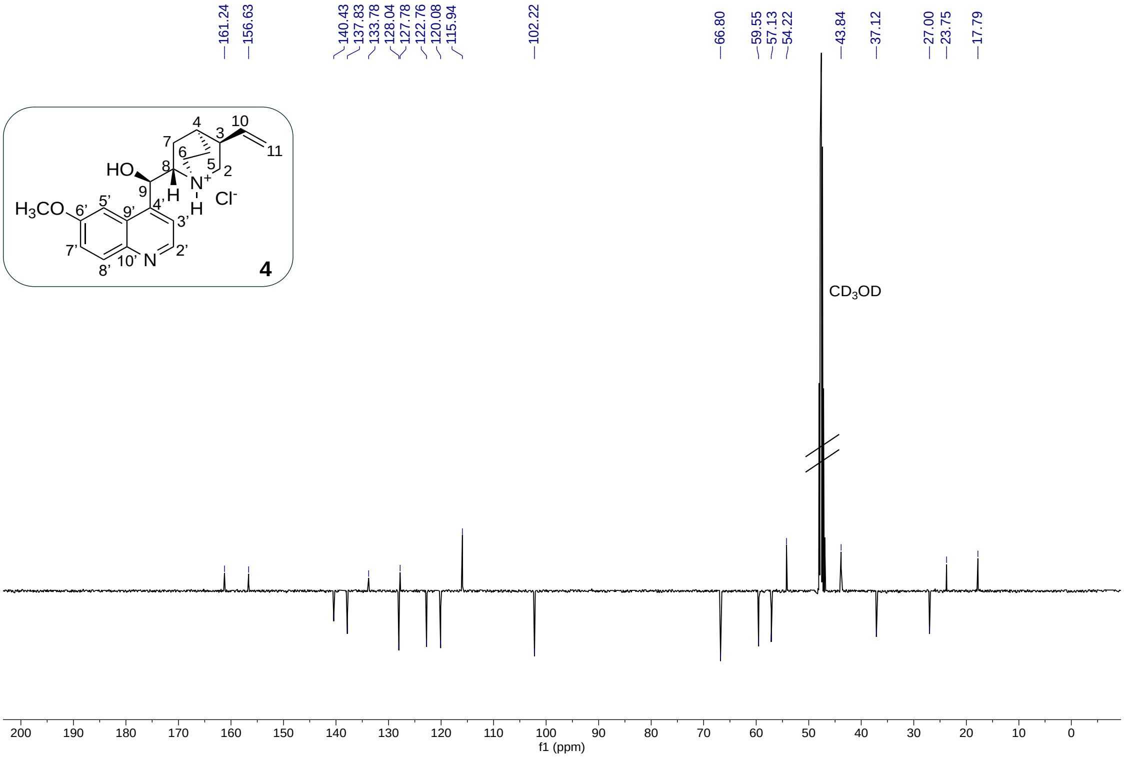

Figure S14. ^{13}C NMR spectrum of quinine hydrochloride (**4**) in CD_3OD.

NMR spectra of oxazolidin-2-ones

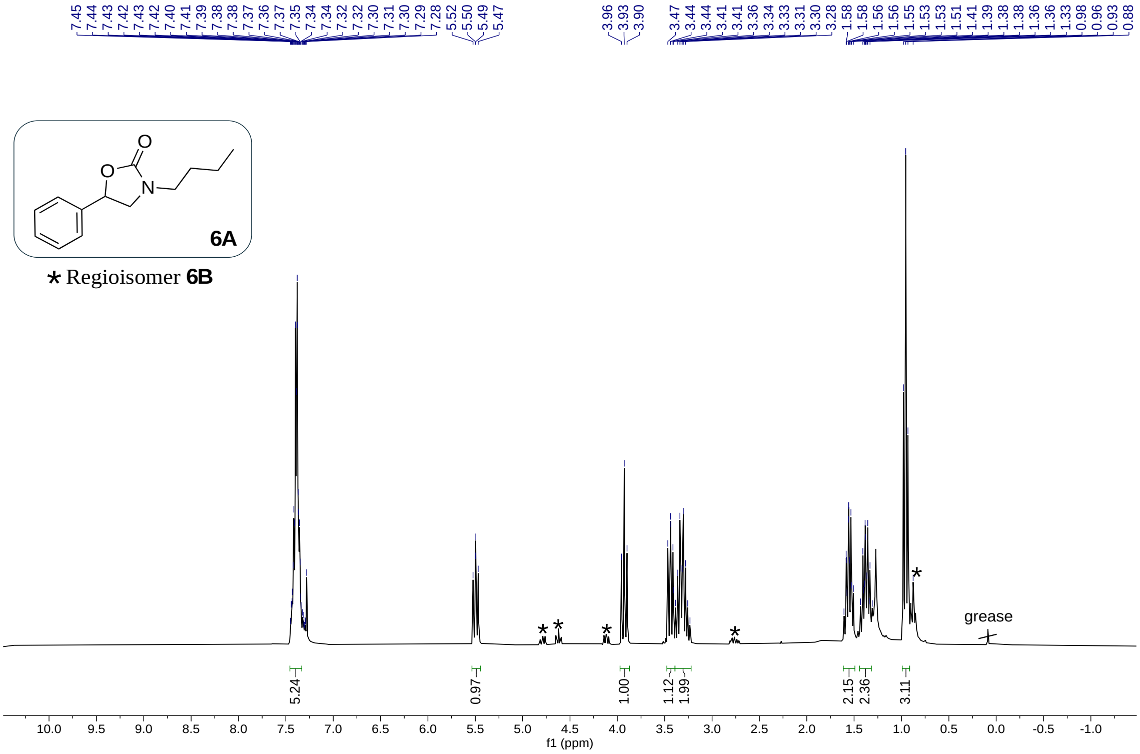

Figure S15. ^{1}H NMR spectrum of 3-butyl-5-phenyloxazolidin-2-one (**6A**) in $CDCl_3$.

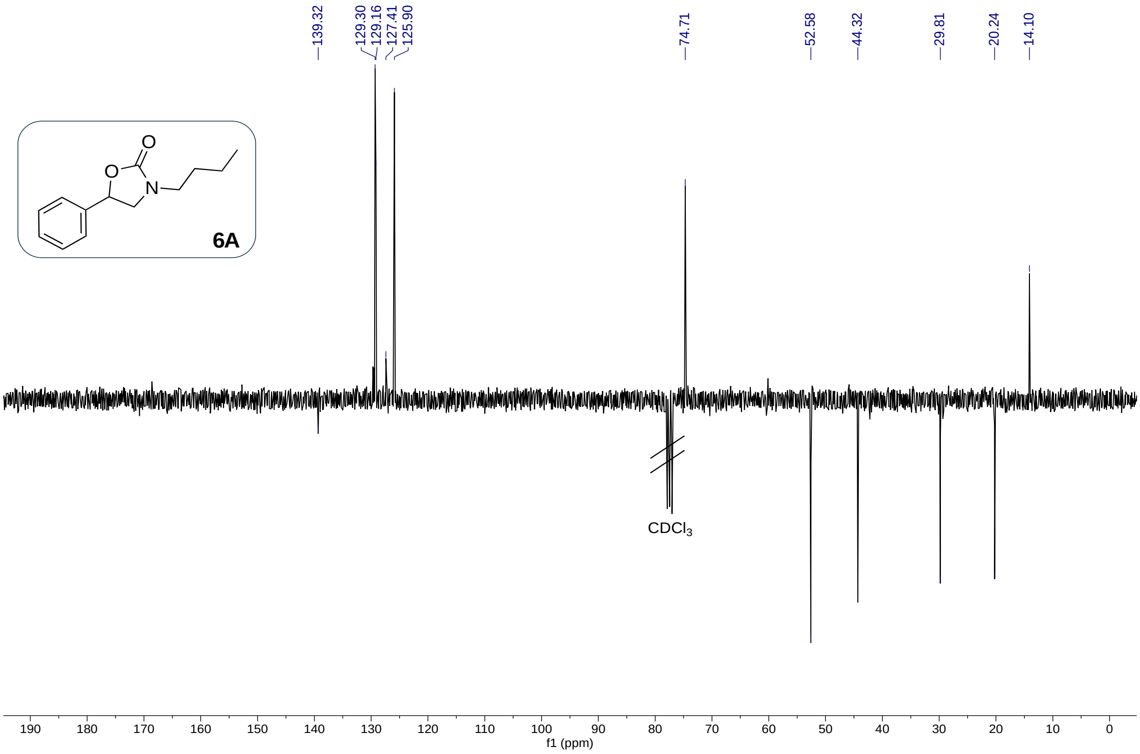

Figure S16. ^{13}C NMR spectrum of 3-butyl-5-phenyloxazolidin-2-one (**6A**) in $CDCl_3$.

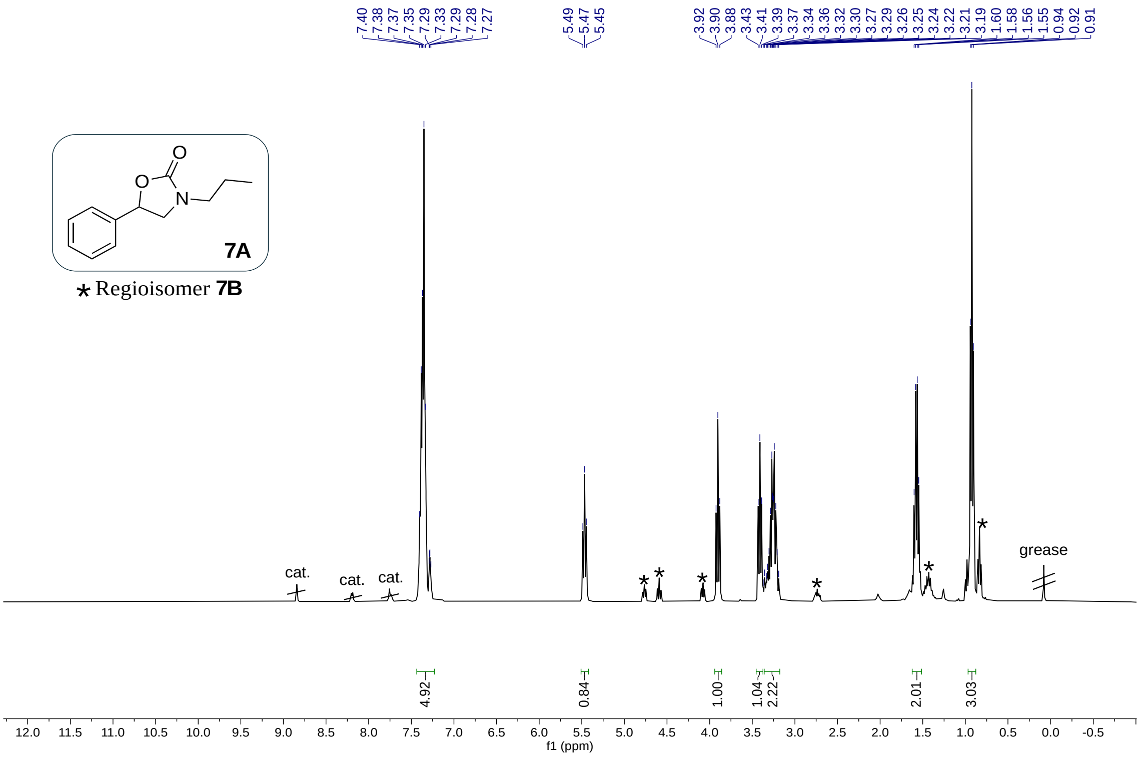

Figure S17. ^{1}H NMR spectrum of 3-propyl-5-phenyloxazolidin-2-one (**7A**) in $CDCl_3$.

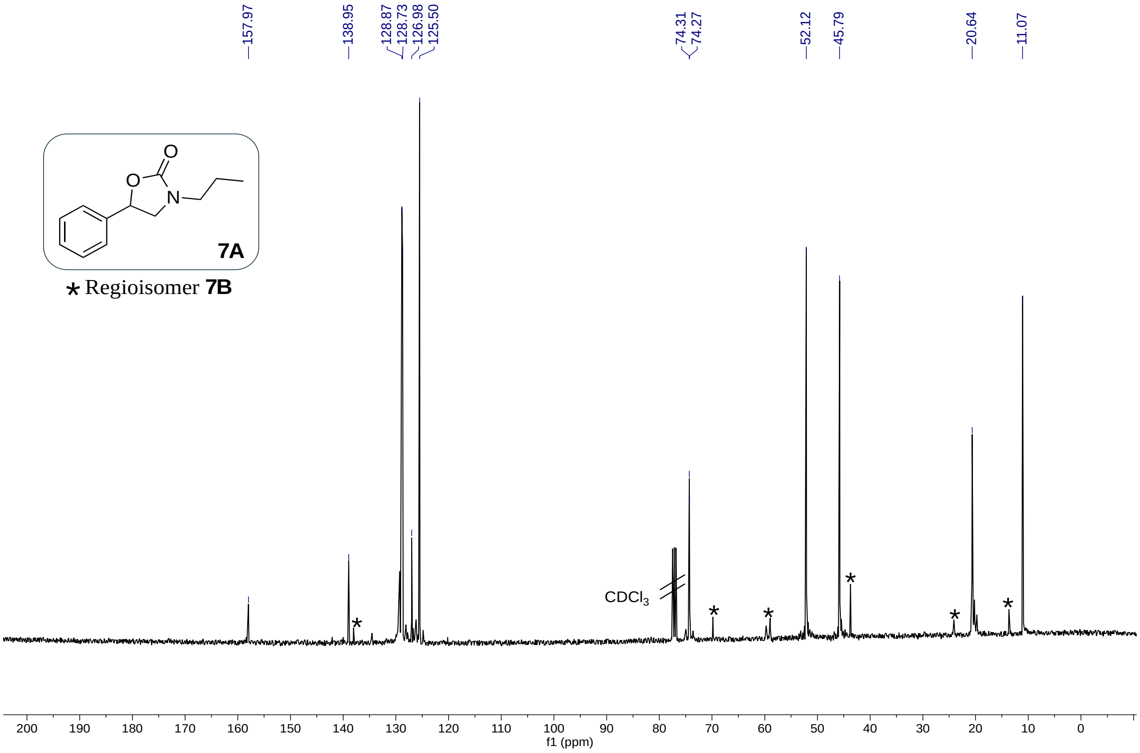

Figure S18. ^{13}C NMR spectrum of 3-propyl-5-phenyloxazolidin-2-one (**7A**) in $CDCl_3$.

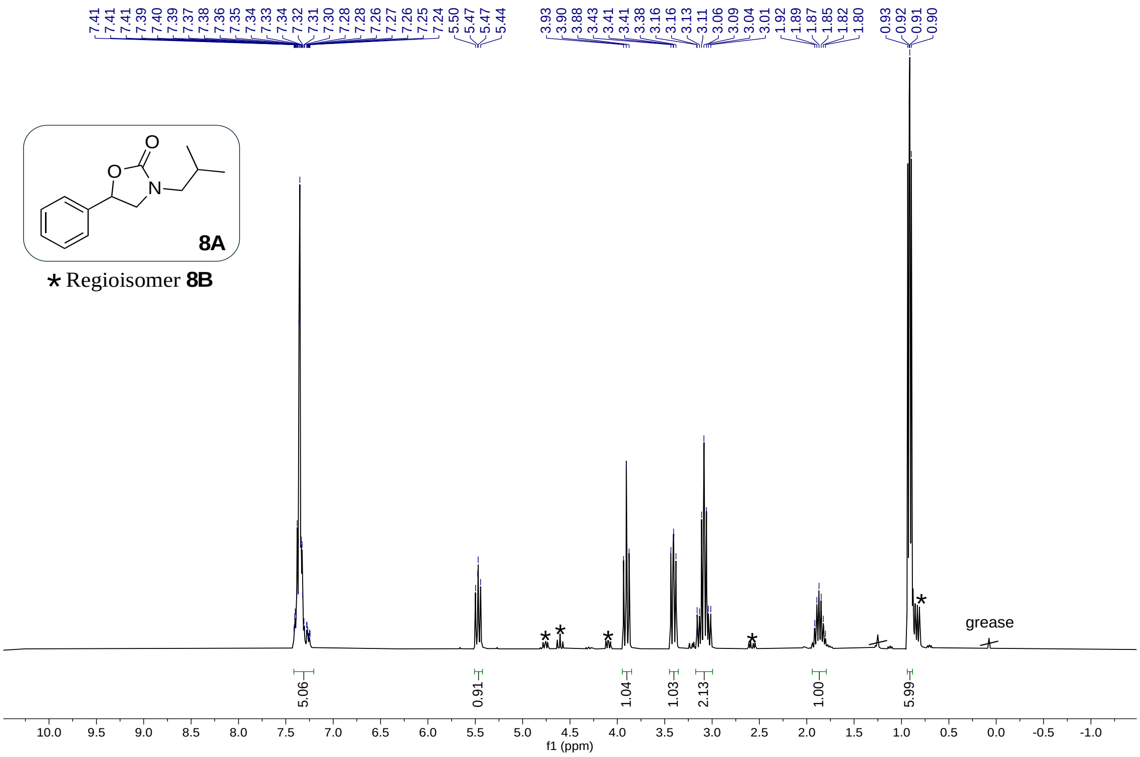

Figure S19. ^{1}H NMR spectrum of 3-isobutyl-5-phenyloxazolidin-2-one (**8A**) in $CDCl_3$.

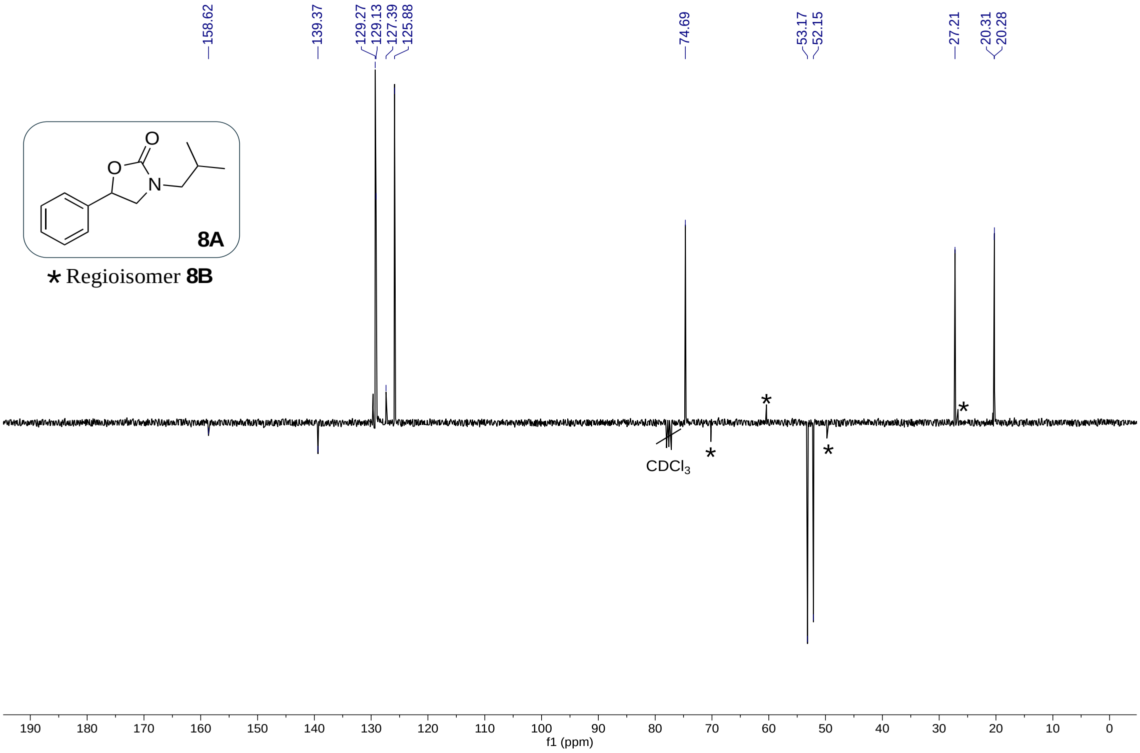

Figure S20. ^{13}C NMR spectrum of 3-isobutyl-5-phenyloxazolidin-2-one (**8A**) in $CDCl_3$.

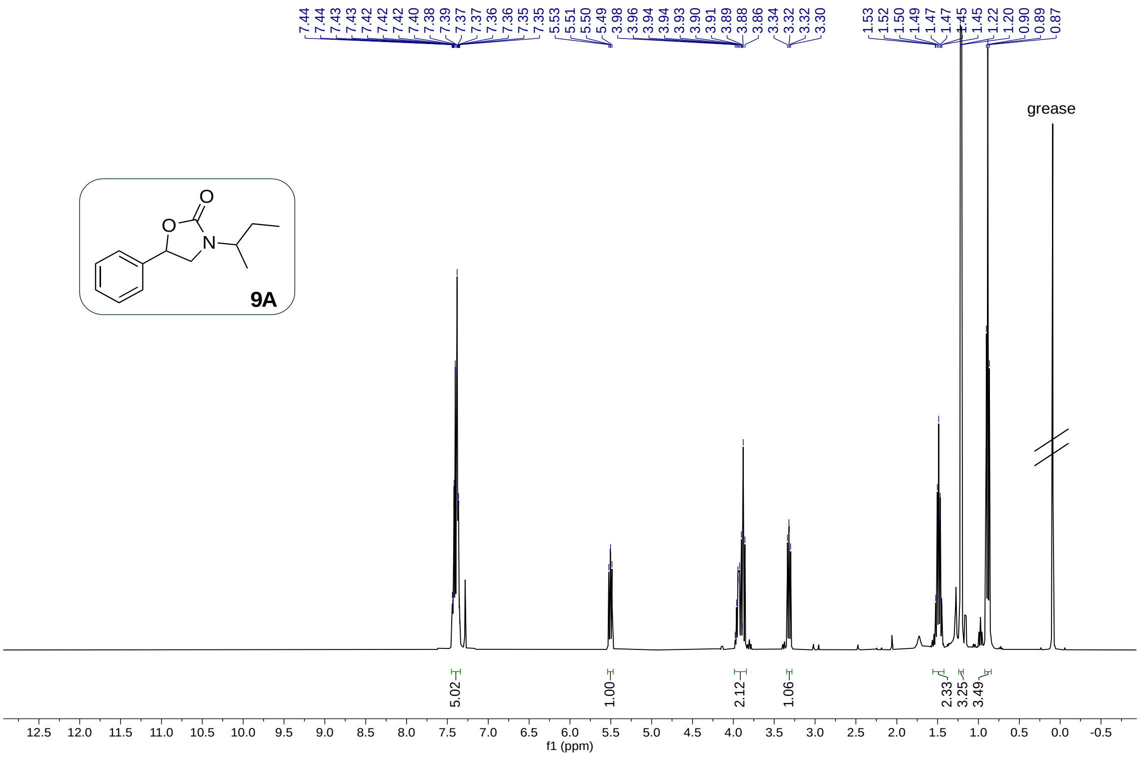

Figure S21. ^{1}H NMR spectrum of 3-secbutyl-5-phenyloxazolidin-2-one (**9A**) in $CDCl_3$.

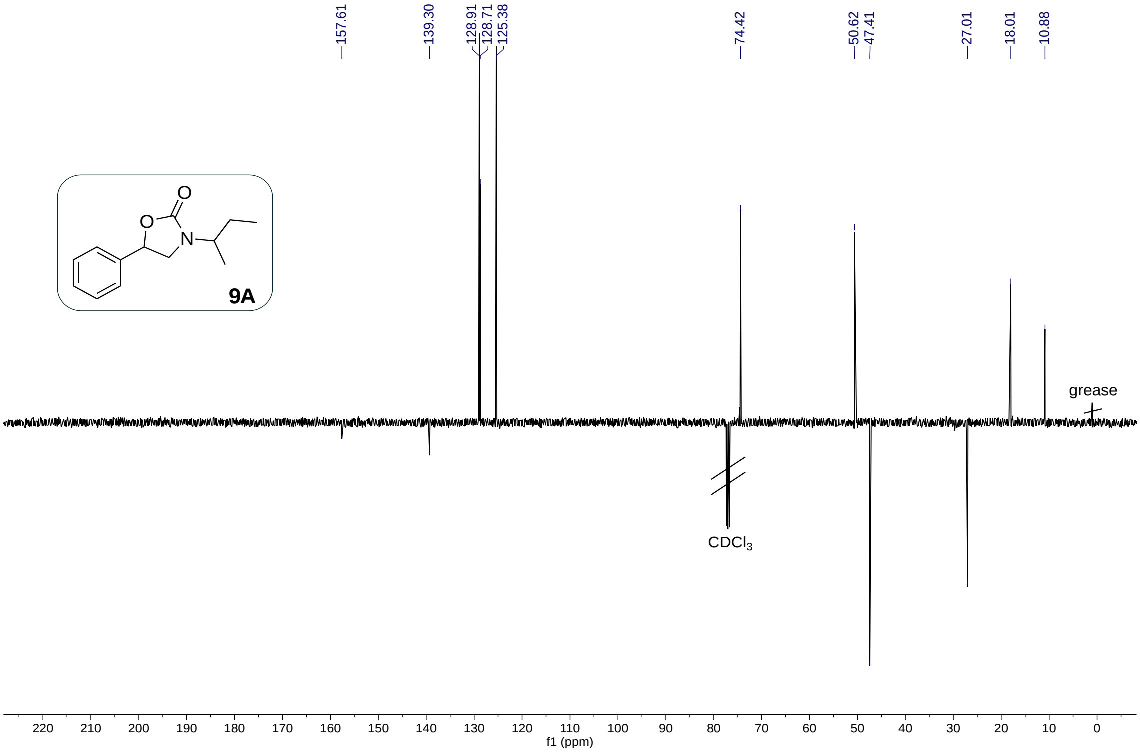

Figure S22. ^{13}C NMR spectrum of 3-secbutyl-5-phenyloxazolidin-2-one (**9A**) in $CDCl_3$.

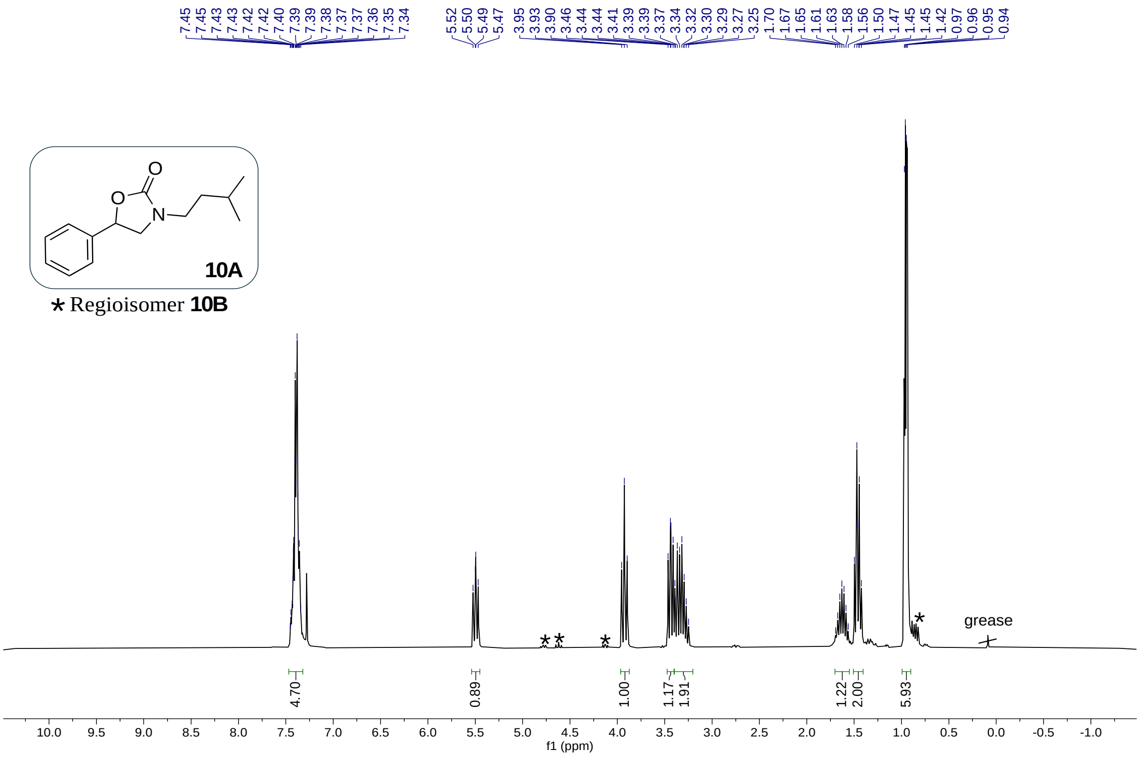

Figure S23. ^{1}H NMR spectrum of 3-isopentyl-5-phenyloxazolidin-2-one (**10A**) in $CDCl_3$.

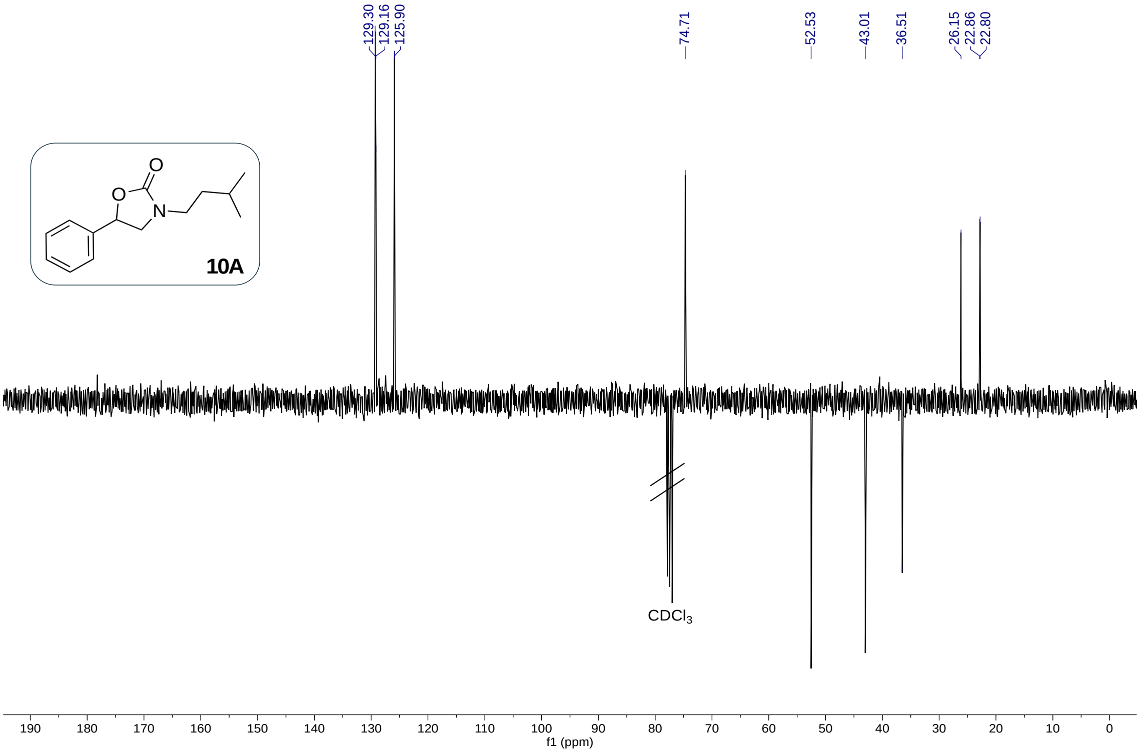

Figure S24. ^{13}C NMR spectrum of 3-isopentyl-5-phenyloxazolidin-2-one (**10A**) in $CDCl_3$.

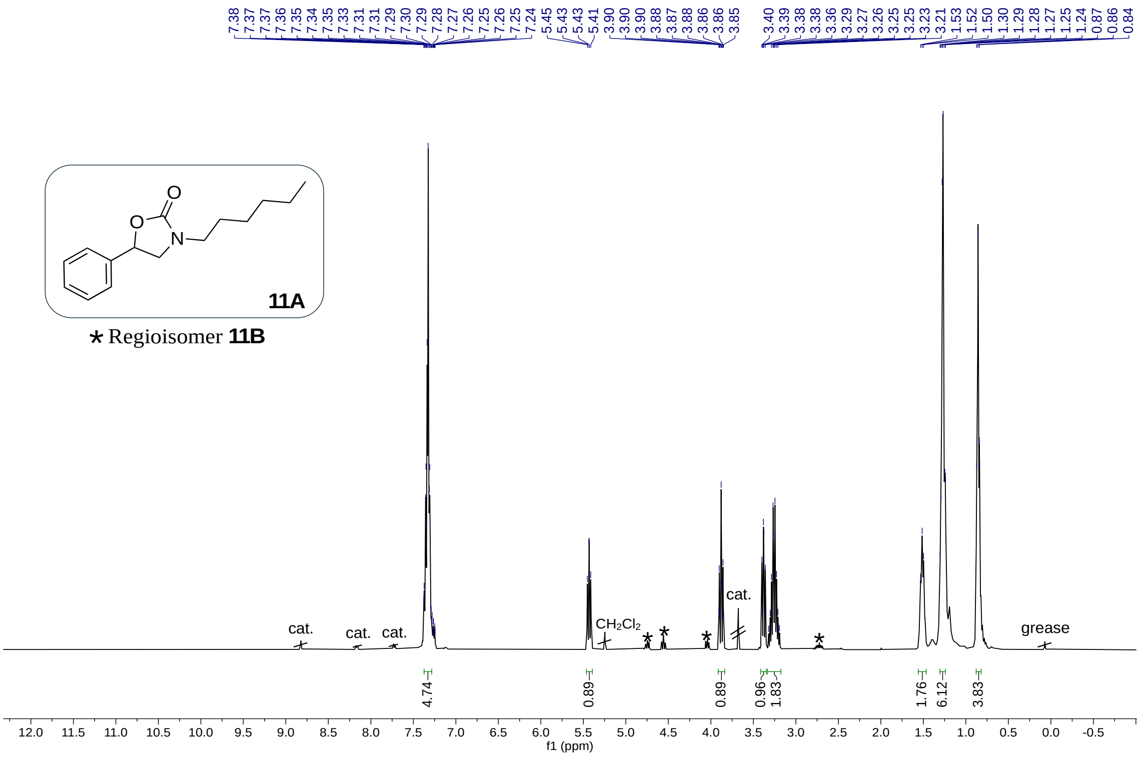

Figure S25. ^{1}H NMR spectrum of 3-hexyl-5-phenyloxazolidin-2-one (**11A**) in $CDCl_3$.

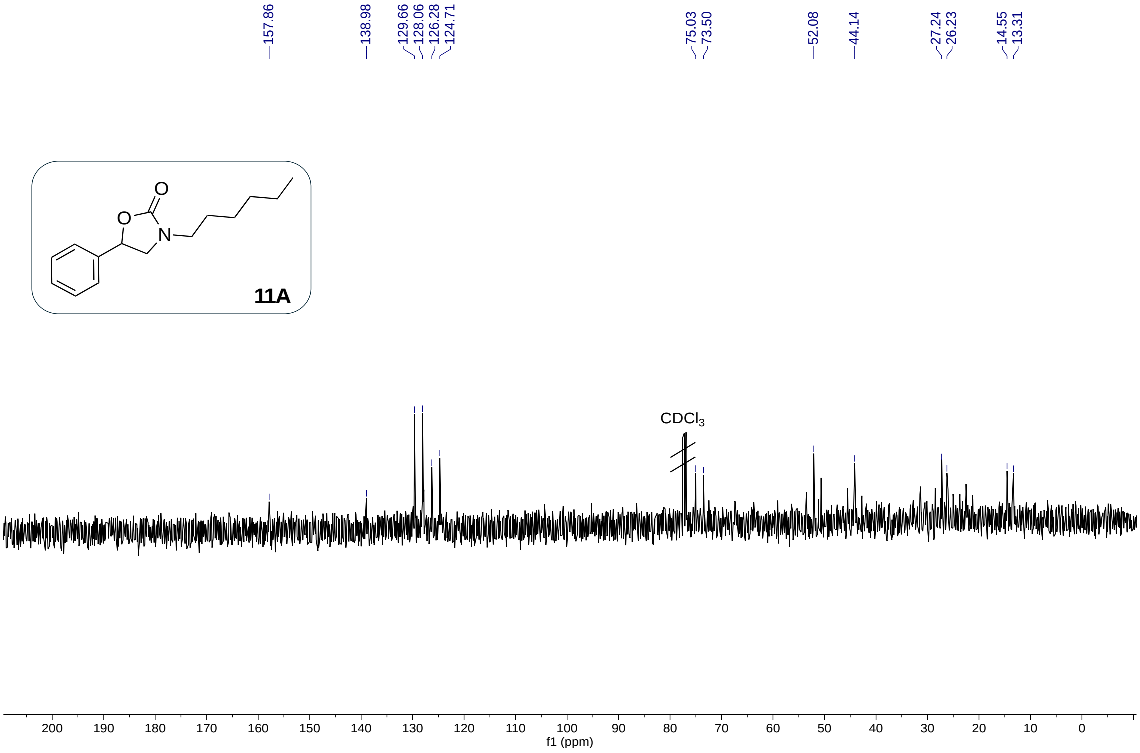

Figure S26. ^{13}C NMR spectrum of 3-hexyl-5-phenyloxazolidin-2-one (**11A**) in $CDCl_3$.

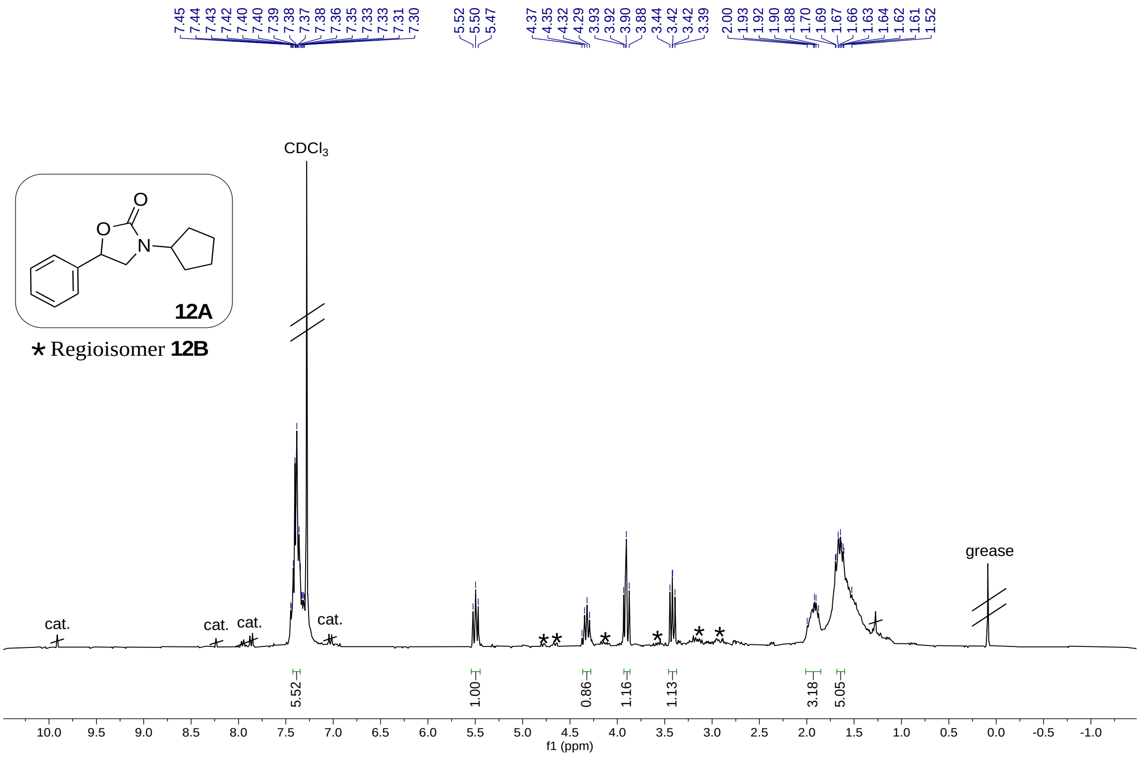

Figure S27. ^{1}H NMR spectrum of 3-cyclopentyl-5-phenyloxazolidin-2-one (**12A**) in $CDCl_3$.

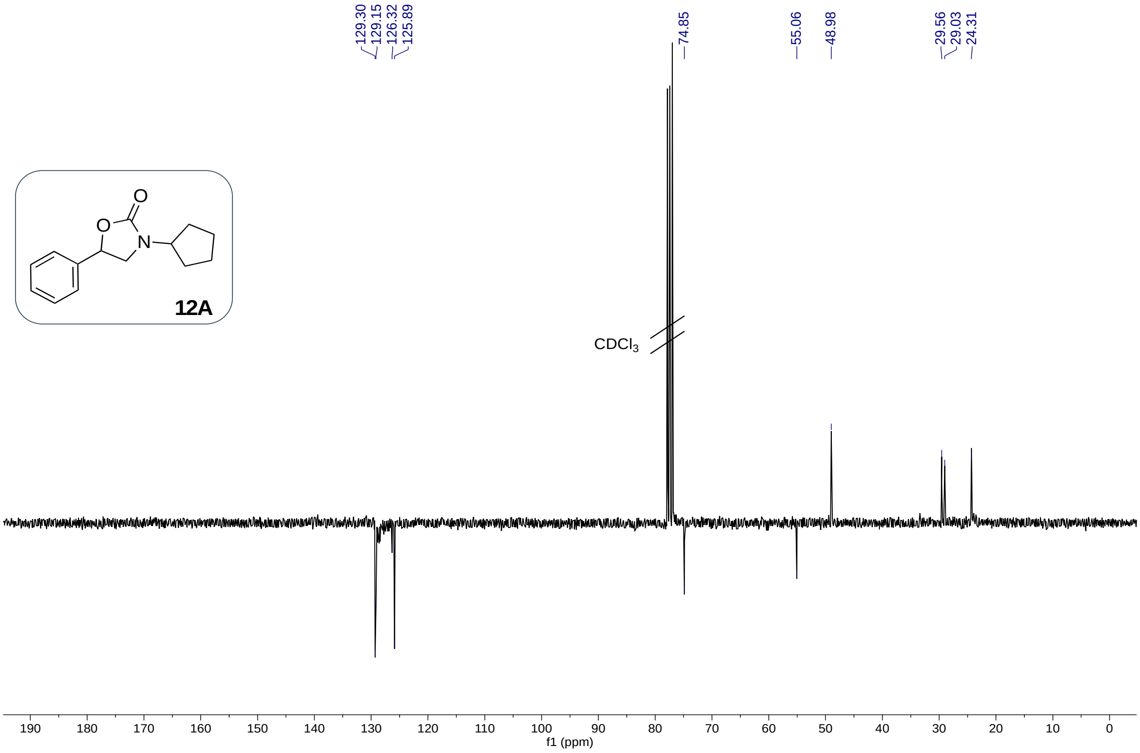

Figure S28. ^{13}C NMR spectrum of 3-cyclopentyl-5-phenyloxazolidin-2-one (**12A**) in $CDCl_3$.

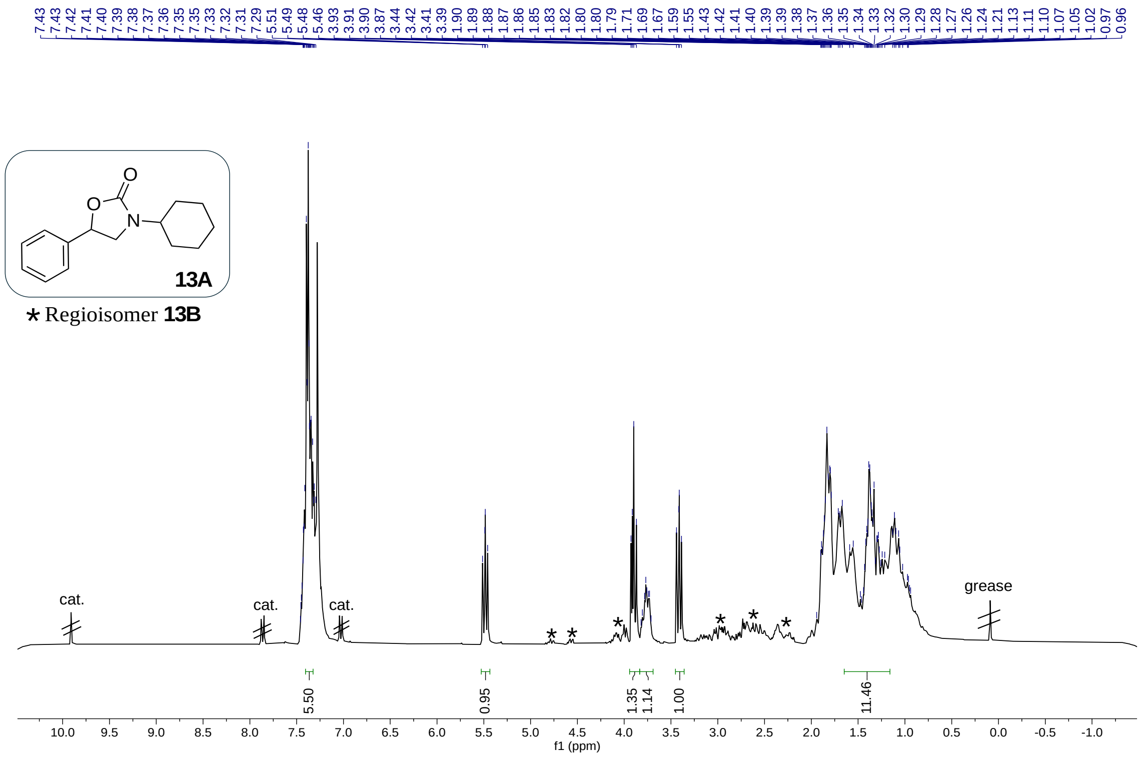

Figure S29. ^{1}H NMR spectrum of 3-cyclohexyl-5-phenyloxazolidin-2-one (**13A**) in $CDCl_3$.

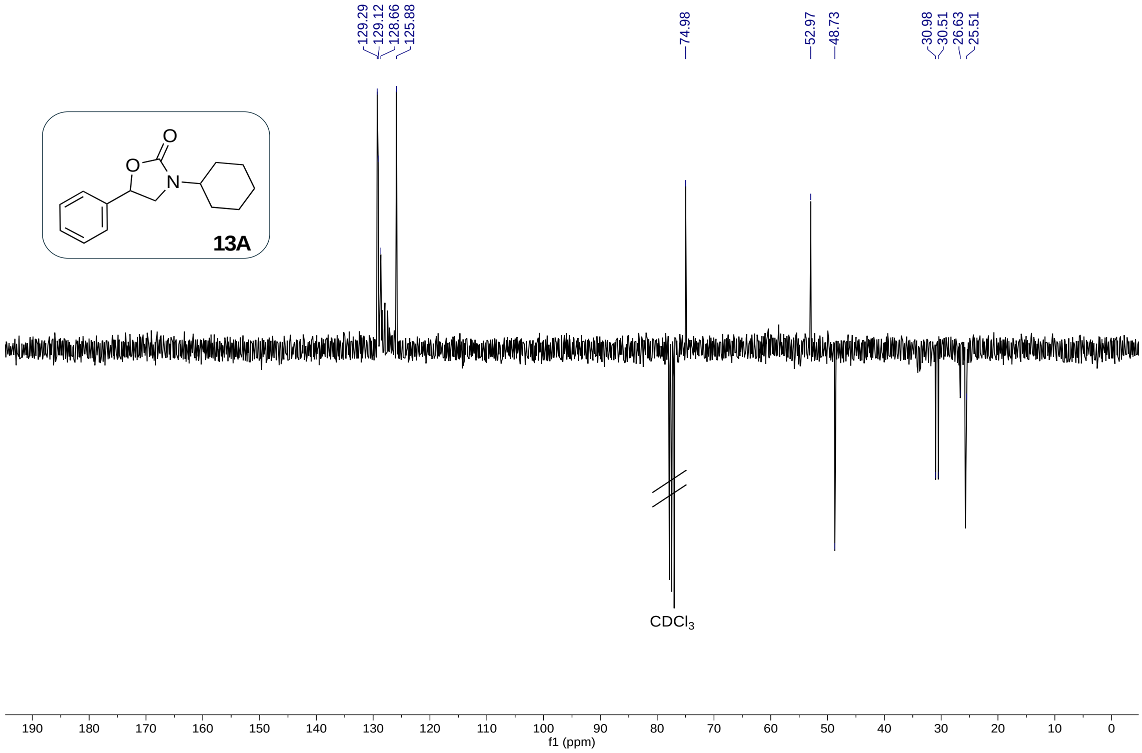

Figure S30. ^{13}C NMR spectrum of 3-cyclohexyl-5-phenyloxazolidin-2-one (**13A**) in $CDCl_3$.

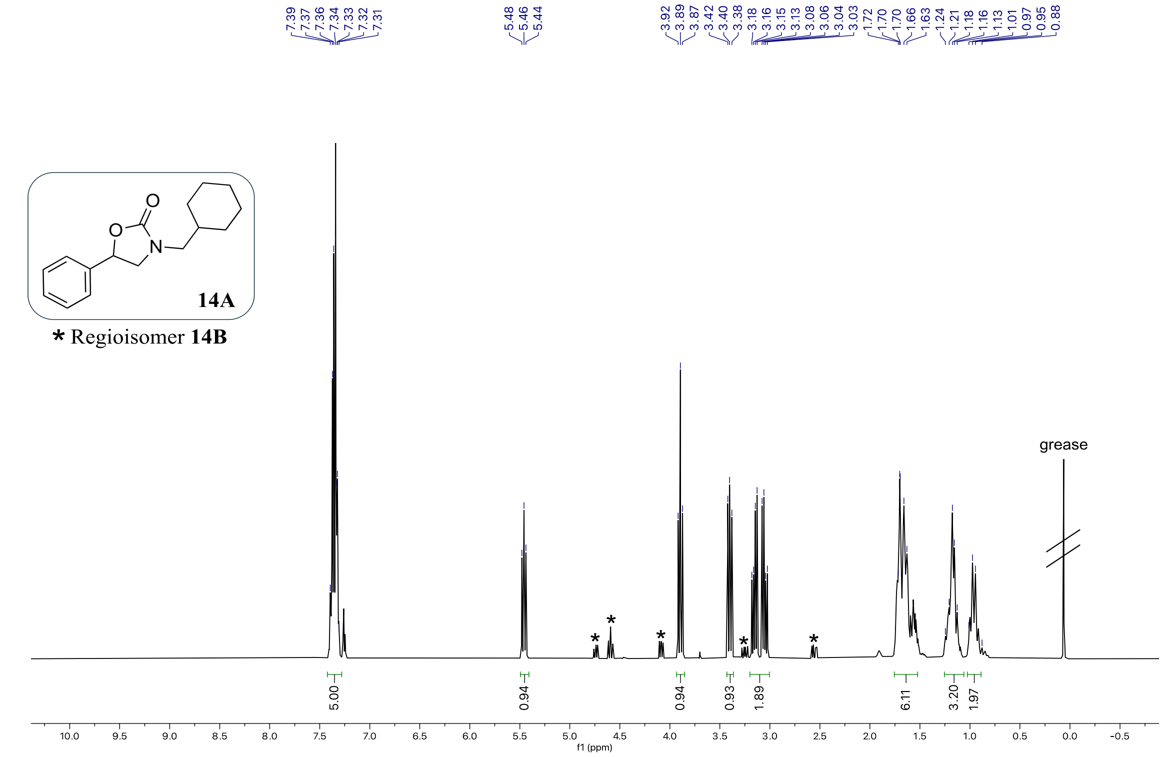

Figure S31. ^{1}H NMR spectrum of 3-cyclohexanemethyl-5-phenyloxazolidin-2-one (**14A**) in $CDCl_3$.

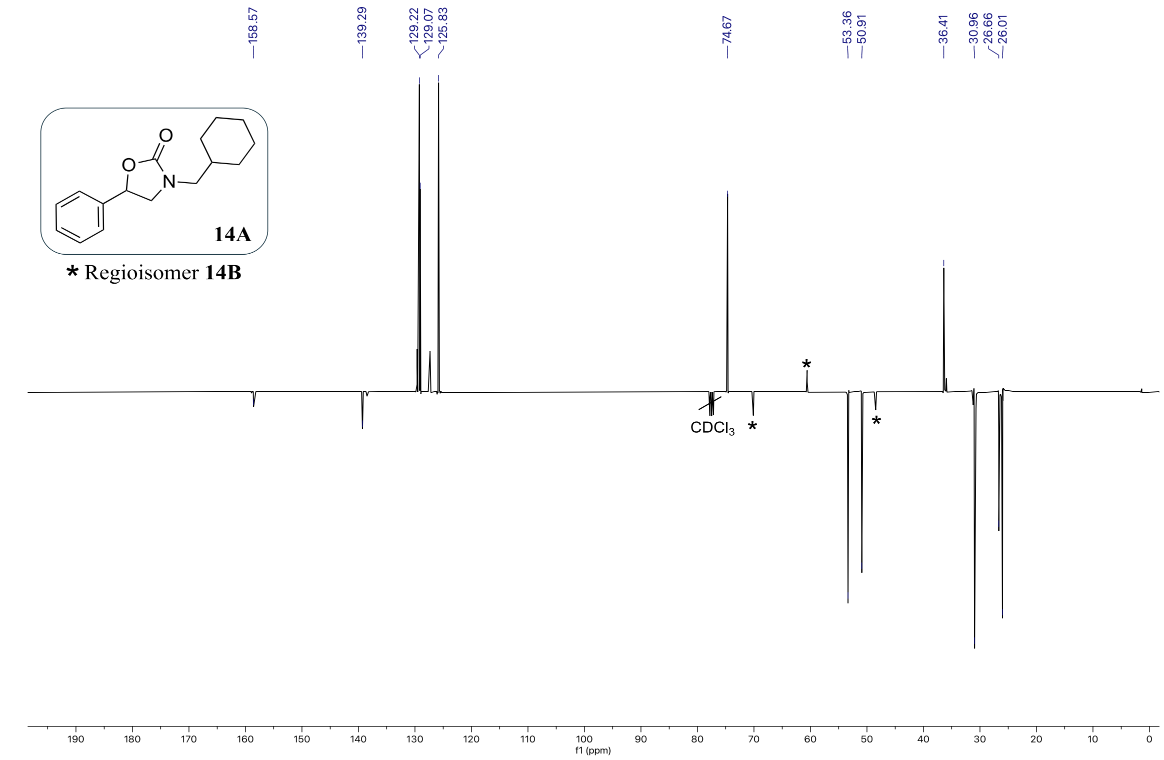

Figure S32. ^{13}C NMR spectrum of 3-cyclohexanemethyl-5-phenyloxazolidin-2-one (**14A**) in $CDCl_3$.

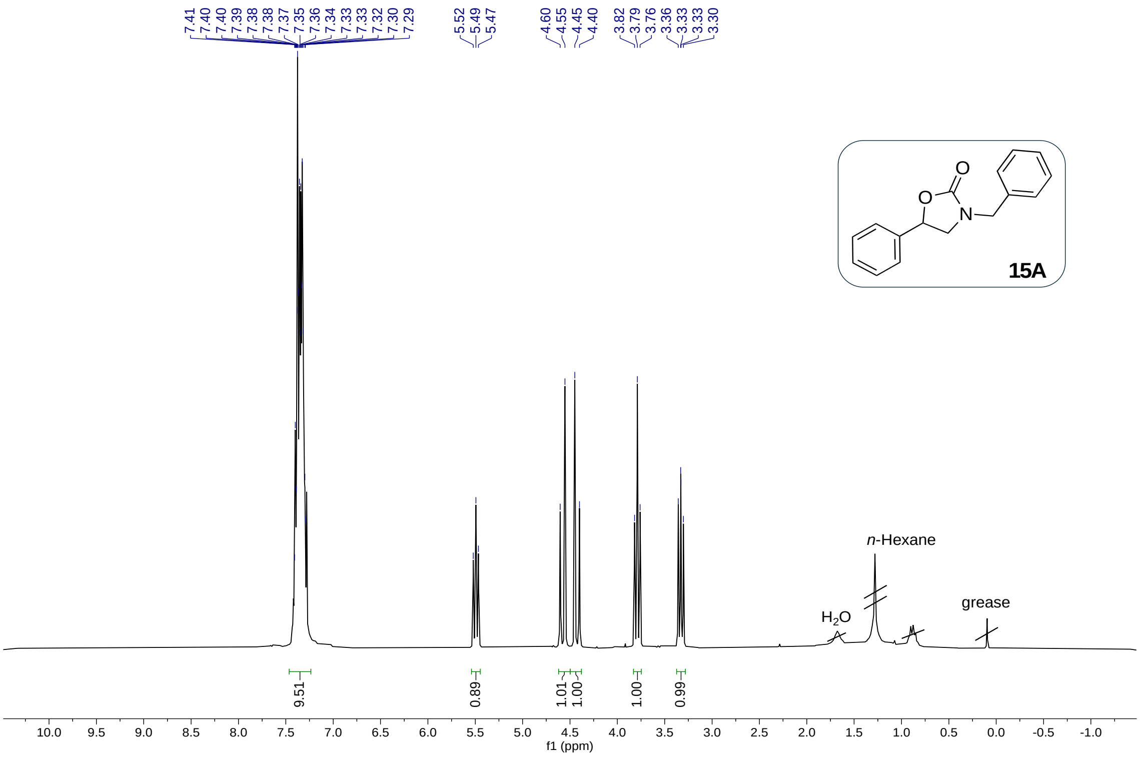

Figure S33. ^{1}H NMR spectrum of 3-benzyl-5-phenyloxazolidin-2-one (**15A**) in $CDCl_3$.

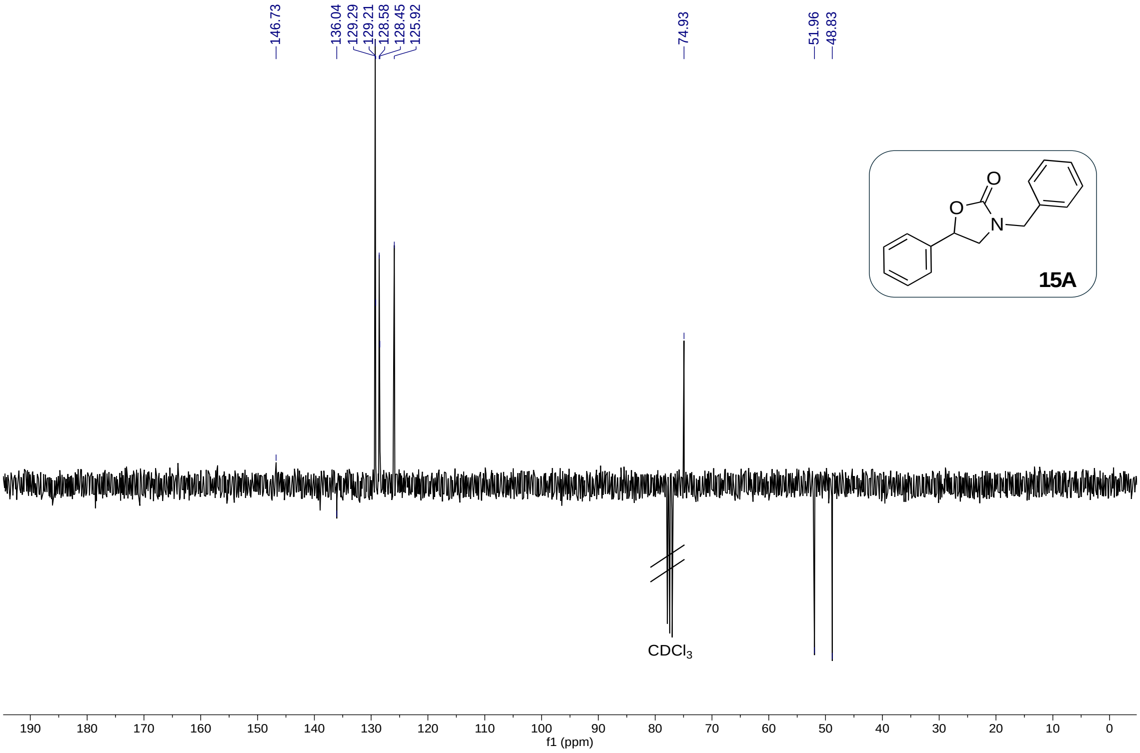

Figure S34. ^{13}C NMR spectrum of 3-benzyl-5-phenyloxazolidin-2-one (**15A**) in $CDCl_3$.

NMR spectra of (*R*)-2-chlorodecan-1-ol

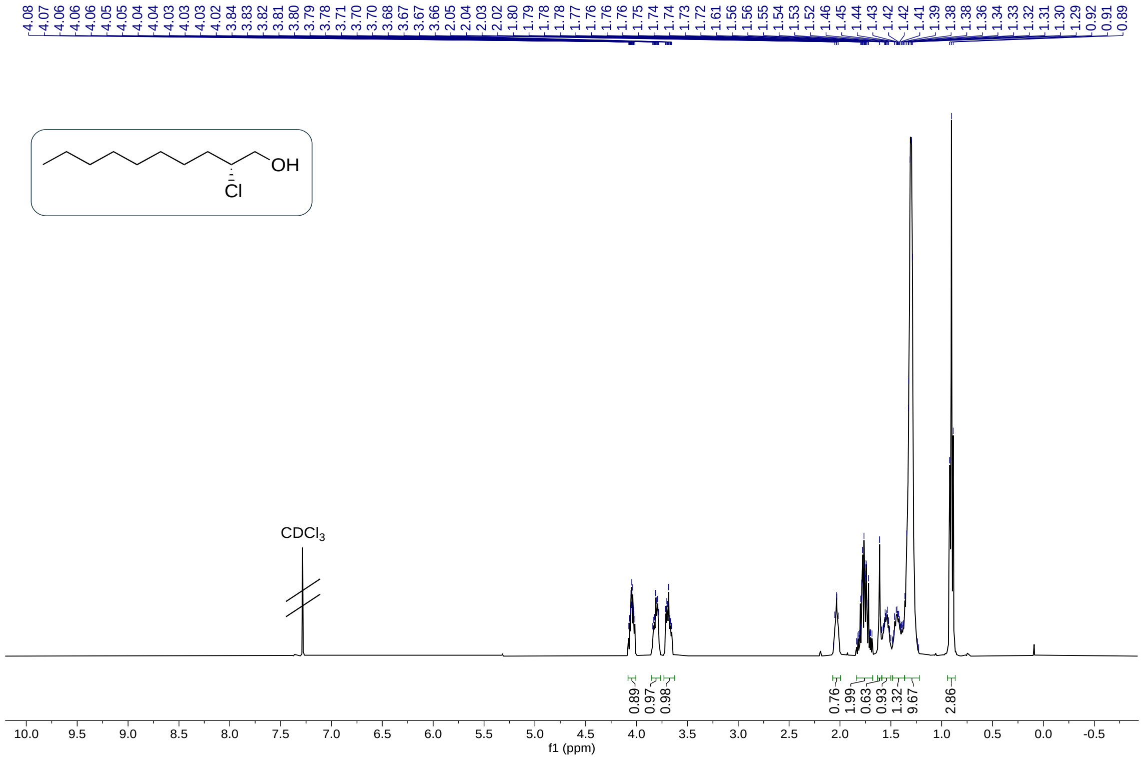

Figure S35. ^{1}H NMR spectrum of (*R*)-2-chlorodecan-1-ol in $CDCl_3$.

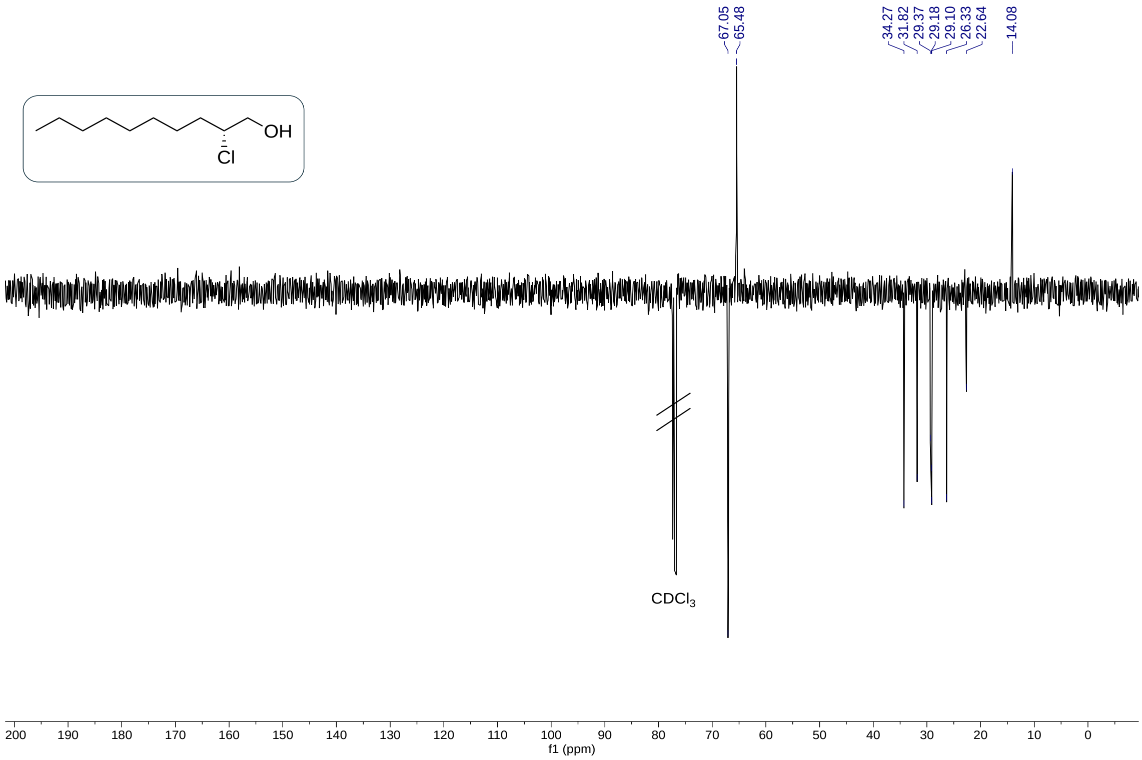

Figure S36. ^{13}C NMR spectrum of (*R*)-2-chlorodecan-1-ol in $CDCl_3$.

NMR spectra of (*S*)-1-benzyl-2-octylaziridine (20)

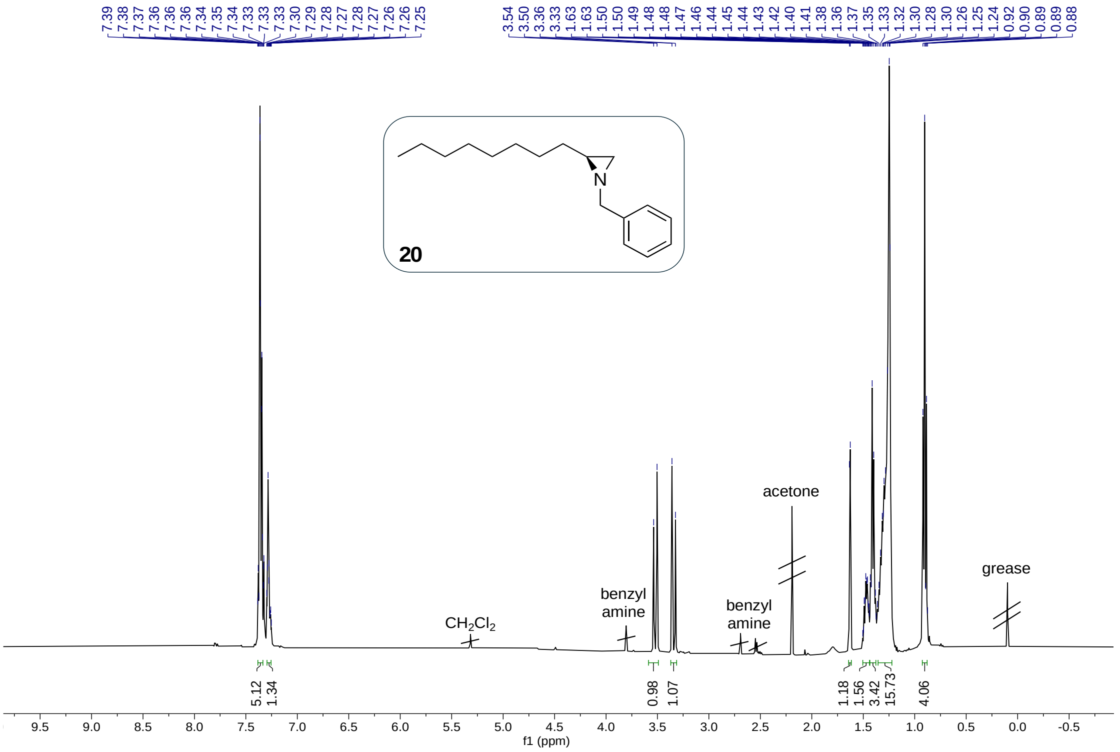

Figure S37. ^{1}H NMR spectrum of (*S*)-1-benzyl-2-octylaziridine (**20**) in $CDCl_3$.

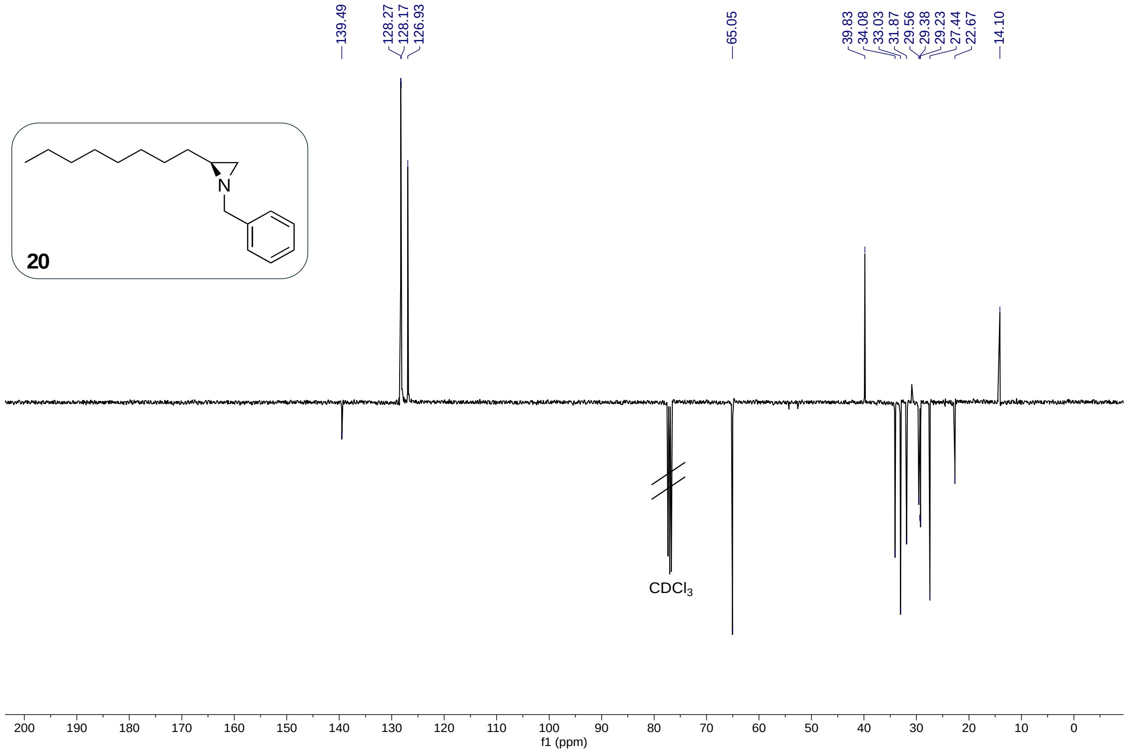

Figure S38. ^{13}C NMR spectrum of (*S*)-1-benzyl-2-octylaziridine (**20**) in $CDCl_3$.

References

[1] Z. Z. Yang, L. N. He, C. X. Miao, S. Chanfreau, *Adv Synth Catal* **2010**, *352*, 2233–2240.

[2] N. Panza, M. Alberti, S. Galiè, C. Damiano, F. Cargnoni, M. Italo Trioni, A. Caselli, *Eur J Org Chem* **2022**, *2022*, e202200908.

[3] T. J. Senter, M. C. O'Reilly, K. M. Chong, G. A. Sulikowski, C. W. Lindsley, *Tetrahedron Lett* **2015**, *56*, 1276–1279.

[4] I. A. Macdonald, *J Chromatogr A* **1977**, *136*, 348–352.

[5] D. Carminati, E. Gallo, C. Damiano, A. Caselli, D. Intrieri, *Eur J Inorg Chem* **2018**, *2018*, 5258–5262.

[6] C. Damiano, P. Sonzini, G. Manca, E. Gallo, *Eur J Org Chem* **2021**, *2021*, 2807–2814.

[7] P. Sonzini, N. Berthet, C. Damiano, V. Dufaud, E. Gallo, *J Catal* **2022**, *414*, 143–154.
